# Glutamate Utilization Couples Oxidative Stress Defense and the Tricarboxylic Acid Cycle in *Francisella* Phagosomal Escape

**DOI:** 10.1371/journal.ppat.1003893

**Published:** 2014-01-16

**Authors:** Elodie Ramond, Gael Gesbert, Mélanie Rigard, Julien Dairou, Marion Dupuis, Iharilalao Dubail, Karin Meibom, Thomas Henry, Monique Barel, Alain Charbit

**Affiliations:** 1 Université Paris Descartes, Sorbonne Paris Cité, Bâtiment Leriche, Paris, France; 2 INSERM, U1002, Unité de Pathogénie des Infections Systémiques, Paris, France; 3 Centre international de recherche en infectiologie, Université de Lyon, Lyon, France; 4 Bacterial Pathogenesis and Innate Immunity Laboratory, INSERM U851 “Immunity, Infection and Vaccination”, Lyon, France; 5 Platform “Bioprofiler” Université Paris Diderot, Paris, France; Emory University School of Medicine, United States of America

## Abstract

Intracellular bacterial pathogens have developed a variety of strategies to avoid degradation by the host innate immune defense mechanisms triggered upon phagocytocis. Upon infection of mammalian host cells, the intracellular pathogen *Francisella* replicates exclusively in the cytosolic compartment. Hence, its ability to escape rapidly from the phagosomal compartment is critical for its pathogenicity. Here, we show for the first time that a glutamate transporter of *Francisella* (here designated GadC) is critical for oxidative stress defense in the phagosome, thus impairing intra-macrophage multiplication and virulence in the mouse model. The *gadC* mutant failed to efficiently neutralize the production of reactive oxygen species. Remarkably, virulence of the *gadC* mutant was partially restored in mice defective in NADPH oxidase activity. The data presented highlight links between glutamate uptake, oxidative stress defense, the tricarboxylic acid cycle and phagosomal escape. This is the first report establishing the role of an amino acid transporter in the early stage of the *Francisella* intracellular lifecycle.

## Author Summary

Intracellular bacterial pathogens have developed a variety of strategies to avoid degradation by the host innate immune defense mechanisms triggered upon phagocytocis. We show here for the first time that glutamate acquisition is essential for phagosomal escape and virulence of an intracellular pathogen. Remarkably, inactivation of the glutamate transporter GadC of *Francisella* impaired the capacity of the bacterium to neutralize reactive oxygen species (ROS) production in the phagosome. Virulence of the *gadC* mutant was partially restored in mice with a defective NADPH oxidase. Importantly, we found that impaired glutamate uptake affected the production of tricarboxylic acid (TCA) cycle intermediates, highlighting novel links between the TCA cycle and bacterial phagosomal escape. Amino acid transporters are, thus, likely to constitute underscored players in microbial intracellular parasitism.

## Introduction

*Francisella tularensis* is a Gram-negative bacterium causing the disease tularemia in a large number of animal species. This highly infectious bacterial pathogen can be transmitted to humans in numerous ways [1], including direct contact with sick animals, inhalation, ingestion of contaminated water or food, or by bites from ticks, mosquitoes or flies. Four different subspecies (subsp.) of *F. tularensis* that differ in virulence and geographic distribution exist, designated subsps. *tularensis*, *holarctica*, *mediasiatica* and *novicida*, respectively. The *tularensis* subspecies is the most virulent causing a severe disease in humans [2], [3]. *F. tularensis* subsp. *novicida* (*F. novicida*) is rarely pathogenic to non-immuno-compromized humans but is fully virulent for mice and is therefore widely used as a model to study *Francisella* intracellular parasitism.

*F. novicida* has the capacity to evade host defenses and to replicate to high numbers within the cytosol of eukaryotic cells [4]. The bacterium is able to enter and to replicate inside a variety of cells, and in particular in macrophages. After a transient passage through a phagosomal compartment, bacteria are released within 30–60 minutes in the host cell cytosol where they undergo several rounds of active replication [1]. Upon *Francisella* entry into macrophages, the phagosomal compartment transiently acidifies and the activation of NADPH oxidase leads to the production of noxious oxygen reactive species [5]. Although several genes required for phagosomal escape have been identified ([6], [7] and references therein), the molecular mechanisms underlying this complex process are still very poorly understood.

Protection against oxidative stress includes the production of anti-oxidant molecules (such as glutathione and NADPH) and of enzymes (such as catalases, superoxide dismutases glutaredoxin-related protein and alkylhydroperoxide reductases). *Francisella* subspecies encode a whole set of such oxidative stress-related enzymes [8]. Inactivation of the corresponding genes generally leads to increased sensitivity to oxidative stress, defective intracellular multiplication, and attenuated virulence [9], [10], [11]. Protection against oxidative and other stress also involves a number of dedicated protein chaperones and chaperone complexes [12].

In contrast, the importance of acid-resistance mechanisms in *Francisella* intracellular survival remains controversial [13], [14], [15] and their possible contribution to pathogenesis still largely unknown. One of the best characterized acid-resistance systems in bacteria couples the glutamate:γ-aminobutyrate exchanger GadC with the glutamate decarboxylase(s) GadA and/or GadB [16]. The decarboxylase replaces the α-carboxyl group of its amino acid substrate with a proton that is consumed from the cytoplasmic pool [17]. The capacity to produce γ-aminobutyric acid (GABA) through glutamate decarboxylation has been observed in both Gram-negative and Gram-positive bacteria. The GadC/GadB glutamate decarboxylase (GAD) system has been shown to play an essential role in acid tolerance in food-borne bacterial pathogens that must survive the potentially lethal acidic environments of the stomach before reaching the intestine. Some bacteria possess a unique permease-decarboxylase pair whereas others, like *Listeria monocytogenes* [18], encode several paralogues of each component.

Recent genome sequence analyses and genome-scale genetic studies suggest that an important proportion of genes related to metabolic and nutritional functions participate to *Francisella* virulence [19]. However, the relationship between nutrition and the *in vivo* life cycle of *F. tularensis* remain poorly understood. *Francisella* is predicted to possess numerous nutrient uptake systems to capture its necessary host-derived nutrients, some of which are probably available in limiting concentrations. Notably, we showed very recently that an asparagine transporter of the major facilitator superfamily of transporters was specifically required for cytoslic multiplication of *Francisella* and its systemic dissemination [20].

The amino acid-polyamine-organocation family of transporters (APC) is specifically involved in amino acid transport [19]. Remarkably, eight of the 11 APC members have been identified at least once in earlier genetic studies, and are likely to be involved in bacterial virulence. In particular, the gene encoding the GadC permease has been identified in several different genome-wide screens, performed in either *F. tularensis* subsp. *holarctica* [21] or *F. novicida* [22], [23], [24].

In the present work, we elucidate the functional role of the GadC protein in *Francisella* pathogenesis. We show that glutamate uptake plays a critical role in *Francisella* oxidative stress defense in the phagosomal compartment. Strikingly, the activity of GadC influences the expression of metabolic genes and the production of tricarboxylic acid (TCA) cycle intermediates, unraveling a relationship between oxidative stress defense, metabolism and *Francisella* virulence.

## Results

### The Gad system of *Francisella*

*F. tularensis* subspecies possess a unique putative GAD system, composed of the antiporter GadC and a decarboxylase GadB (encoded by genes *FTN_0571* and *FTN_1701* in *F. novicida* and hereafter designated *gadC* and *gadB*, respectively for simplification) (**Figure S1A**). The transcription of *gadC* is initiated 27 nucleotides upstream of the translational start from a predicted σ^70^ promoter (**Figure S1B**). This genetic organization is highly conserved in all the available *F. tularensis* genomes (not shown). The gene *gadC* encodes a protein of 469 amino acids sharing 98.7%, 99.1% and 99.6% identity with its orthologues in the subspecies *mediasiatica* (*FTM_*1423), *holartica* (*FTL_1583*) and *tularensis* (*FTT_0480c*), respectively.

The *Francisella* GadC protein is predicted as a putative glutamate:γ-aminobutyric acid (GAD) antiporter (KEGG database). Although it shows only modest homology (approximately 25% amino acid identity) with GadC of *E. coli* [25], secondary structure prediction (using the method for prediction of transmembrane helices HMM available at the internet site www.cbs.dtu.dk) indicates that the GadC transporter of *Francisella* also comprises 12 transmembrane helixes and has its N and C-terminal ends facing the cytoplasm (not shown).

The *gadB* gene encodes a putative glutamate decarboxylase protein of 448 amino acid residues that is highly conserved in *F. tularensis* subsp. *tularensis* (98.7% amino acid identity with FTT_1722c). However, the corresponding protein is truncated at its C-terminal end in the subspecies *holarctica* (FTL_1863) and *mediasiatica* (FTM_1673, and noted as a pseudogene in the KEGG database).

### Role of GadC in bacterial intracellular multiplication and virulence

We constructed a strain with chromosomal deletion of the entire *gadC* gene in *F. novicida* by allelic replacement [26]. We confirmed that the Δ*gadC* mutation did not have any polar effect on the downstream gene *FTN_0570* by quantitative qRT-PCR (**Figure S1C**). The growth kinetics of the parental *F. novicida* strain and the Δ*gadC* mutant were indistinguishable in tryptic soya broth (TSB) and chemically defined medium (CDM) [27] liquid media at 37°C (**Figure S2**), indicating that inactivation of *gadC* had no impact on bacterial growth in broth.

**Figure 1:**
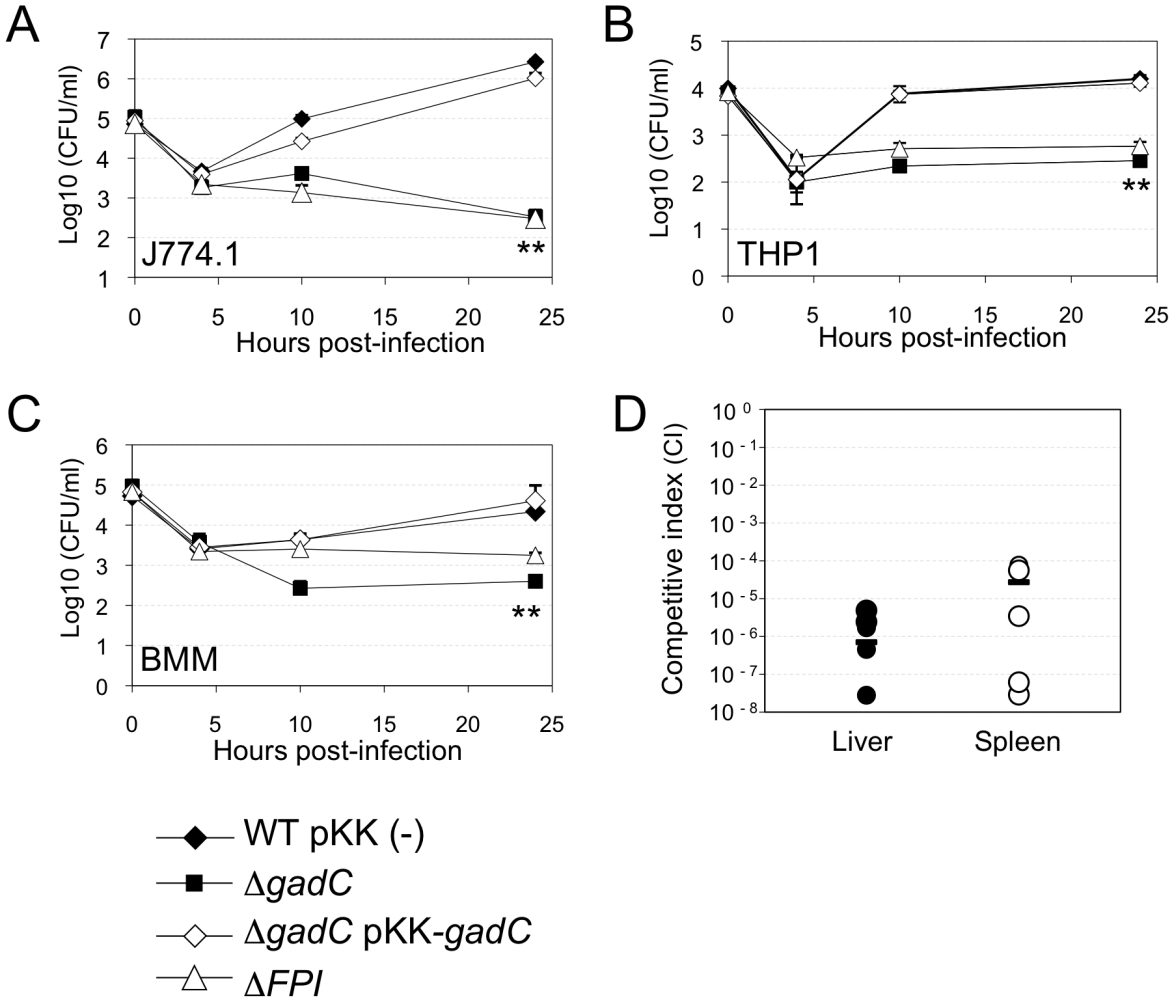
*gadC* inactivation affects intracellular survival and virulence. Intracellular replication of wild-type *F. novicida* (WT) carrying the empty plasmid pKK214 (WT/pKK(−)), of the *ΔgadC* mutant (Δ*gadC*) and complemented strain (Δ*gadC*/pKK-*gadC*), and of the Δ*FPI* mutant (Δ*FPI*), was monitored in J774.1 macrophage-like cells (**A**); in THP-1 human macrophages (**B**); and in bone marrow-derived macrophages (**C**), over a 24 h-period. Results are shown as the average of log_10_ cfu mL^−1^ ± standard deviation. Each experiment was performed in triplicate. **, *p*<0.01 (as determined by the Student's *t*-test). Competition assays (**D**). A group of five female BALB/c mice were infected i.p. with a 1∶1 mixture of wild-type *F. novicida* and Δ*gadC* mutant strains (100 colony forming units (cfu) of each). The data represent the competitive index (CI) value for cfu of mutant/wild-type in the liver (L: black diamonds, left column) and spleen (S: black circles, right column) of each mouse, 48 h after infection. Bars represent the geometric mean CI value.

We examined the ability of wild-type *F. novicida*, the Δ*gadC* mutant and a Δ*gadC* mutant strain complemented with a plasmid-encoded copy of wild-type *gadC*, to survive in murine and human macrophage cell lines and primary bone marrow-derived mouse macrophages, over a 24 h-period. The Δ*gadC* mutant showed a severe growth defect in J774.1 cells, comparable to that of a mutant deleted of the entire *Francisella* pathogenicity island (Δ*FPI* mutant), with more than a 30-fold reduction of intracellular bacteria after 10 h and a 1,000-fold reduction after 24 h (**Figure 1A**). Impaired multiplication of the Δ*gadC* mutant was also observed in THP-1 macrophages (**Figure 1B**) as well as in bone marrow-derived macrophages (**Figure 1C**). In all cell types tested, introduction of the complementing plasmid (pKK*-gadC*) restored bacterial viability to same level as in the wild-type parent, confirming the specific involvement of the *gadC* gene in intracellular survival.

Next, *in vivo* competition assays in BALB/c mice were performed to determine if the GadC protein played a role in the ability of *Francisella* to cause disease. Five mice (6- to 8-week old) were inoculated by the intraperitoneal (i.p.) route with a 1∶1 mixture of wild-type *F. novicida* and Δ*gadC* mutant strains. Bacterial multiplication in the liver and spleen was monitored at day 2 post-infection (**Figure 1D**). The Competition Index (CI), calculated for both organs, was extremely low (10^−6^) demonstrating that the gene *gadC* played an essential role in *Francisella* virulence in the mouse model.

**Figure 2:**
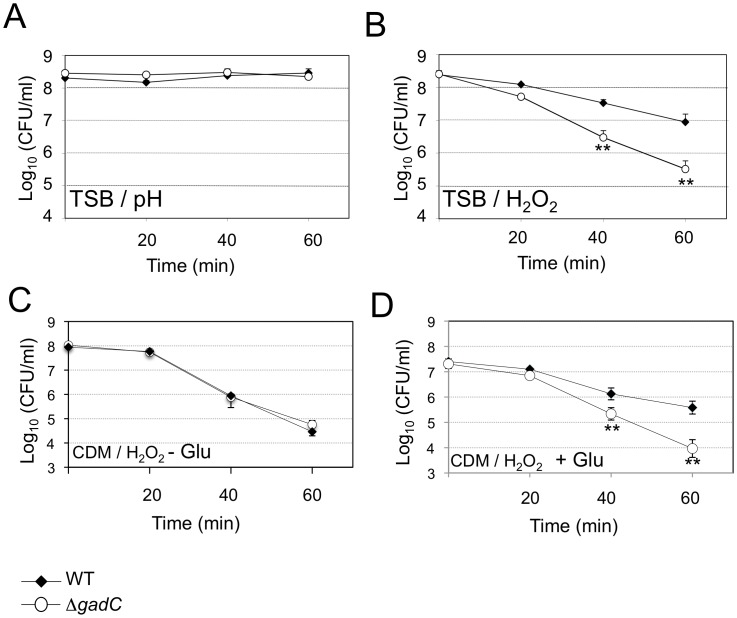
Stress sensitivity. Exponential phase bacteria, diluted in TSB medium, were subjected: (**A**) to acidic stress (pH 5.5), or (**B**) to oxidative stress (500 µM H_2_0_2_). Exponential phase bacteria, diluted in chemically defined medium (CDM) (**C**), or CDM supplemented with 1 mM glutamate (**D**), were subjected to oxidative stress (500 µM H_2_0_2_). The bacteria were plated on chocolate agar plates at different times and viable bacteria were monitored 2 days after. Data are the average cfu mL^−1^ for three points. Experiments were realized twice. **, *p*<0.01 (as determined by the Student's *t*-test).

Upon *Francisella* entry into cells, *Francisella* initially resides in a phagosomal compartment that transiently acidifies and that acquires reactive oxygen species. We therefore examined the ability of wild-type and Δ*gadC* mutant strains to survive under acid or oxidative stress conditions. For this, bacteria were exposed either to pH 5.5 or to 500 µM H_2_O_2_ (**Figure 2**). Under the pH condition tested, the viability of two strains was unaffected (**Figure 2A**). It should be noted that at the lower pH of 2.5, the viability of both wild-type and Δ*gadC* mutant was equally reduced (approximately 2 logs, not shown). In contrast, the Δ*gadC* mutant strain appeared to be significantly more sensitive to oxidative stress than the wild-type strain in TSB (**Figure 2B**). After 40 min of exposure, it showed a 10-fold decrease in the number of viable bacteria and an approximately 50-fold decrease after 60 min of exposure to H_2_O_2_. Remarkably, in CDM, the wild-type and Δ*gadC* mutant strains were equally sensitive to H_2_O_2_ in the absence of glutamate supplementation (**Figure 2C**). However, upon glutamate supplementation, the wild-type strain showed increased resistance to H_2_O_2_ whereas the Δ*gadC* strain was unaffected (**Figure 2D**).

### GadC is involved in phagosomal escape

Confocal and electron microscopy analyses demonstrated that the Δ*gadC* mutant had lost the capacity to escape from the phagosomal compartment of infected macrophages.

#### Confocal microscopy

**Figure 3:**
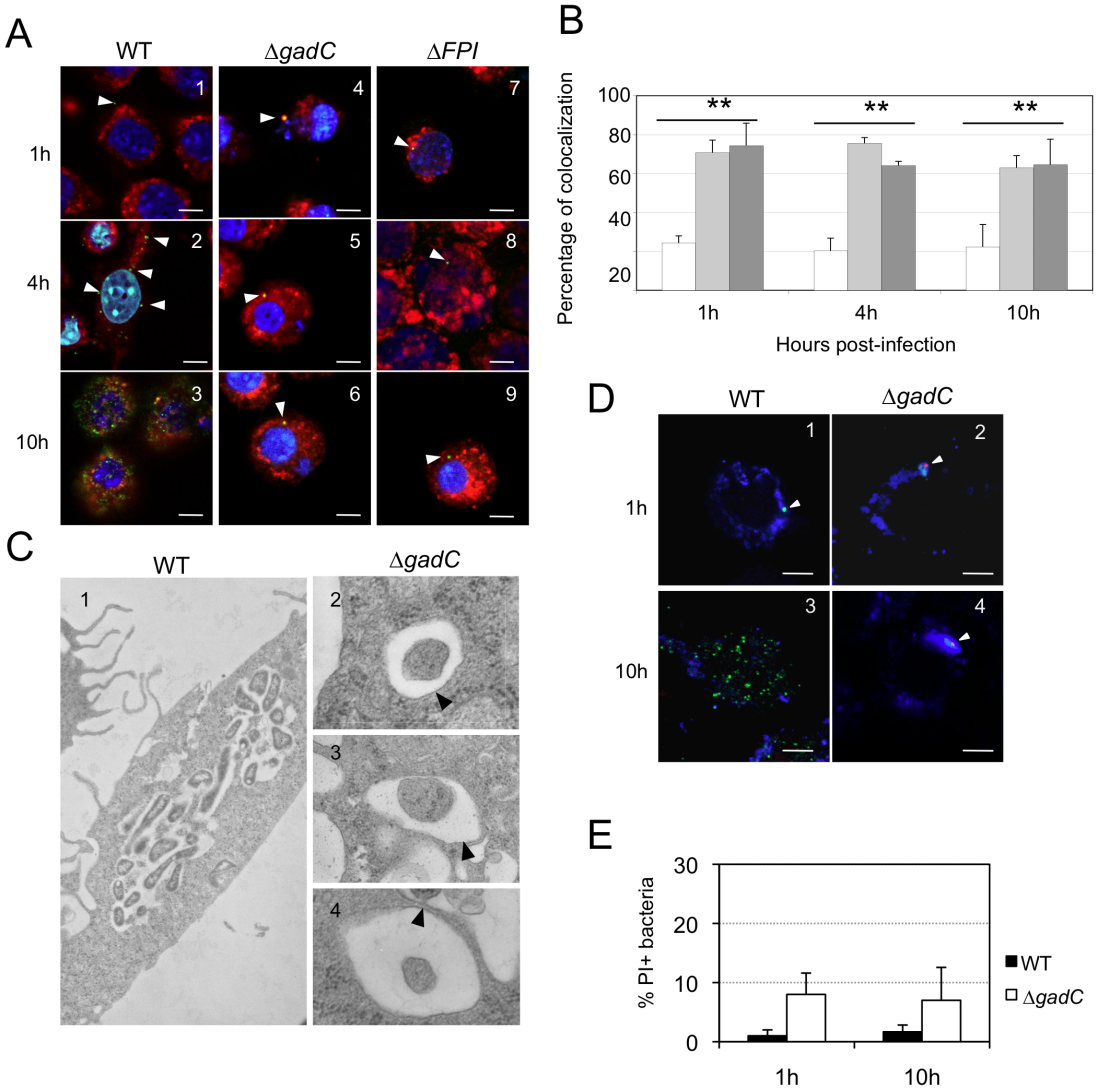
Subcellular localization of the Δ*gadC* mutant. (**A**) Co-localization of wild-type *F. novicida* (**1, 2, 3**), Δ*gadC* (**4, 5, 6**) or Δ*FPI* mutant strain (**7, 8, 9**) with LAMP-1 was monitored by confocal microscopy, in J774.1 macrophage cells. Co-localization was monitored at 1 h (**1, 4, 7**), 4 h (**2, 5, 8**) and 10 h (**3, 6, 9**). Anti-*Francisella* antibody was used at a final dilution of 1∶500 and is shown in green. Anti-LAMP-1 antibody was used at a final dilution of 1∶100 and is shown in red. White arrowheads point to individual bacteria. Cell nuclei were labeled with DAPI (in blue). The images are representative from triplicate coverslips in three independent experiments. Scale bars at the bottom right of each panel correspond to 10 µM. (**B**) Quantification of co-localization between bacteria and LAMP-1 was obtained with Image J software. The graph results from the analysis of 4 different fields for each time of infection, in three independent experiments. **, p<0.01 (as determined by the Student's *t*-test). White bars, *F. novicida* U112 (WT); light grey bars, Δ*gadC*; dark grey bars, Δ*FPI*. (**C**) Transmission electron micrographs of thin sections of J774.1 macrophages, infected by wild-type *F. novicida* and Δ*gadC* mutant strains. Infections were monitored over a 10 h-period. At 10 h, active cytosolic multiplication of wild-type *F. novicida* was observed in most of the infected cells (**1**) whereas the Δ*gadC* mutant remains trapped into spacious phagosomes (**2, 3, 4**). Black arrowheads point to intact phagosomal membrane. (**D**) To evaluate the viability of intracellular *Francisella*, labeling with the cell-impermeant nucleic acid dye propidium iodide (PI) was performed. Confocal images of J774.1 cells, infected with wild-type *F. novicida* (**1, 3**) or Δ*gadC* mutant (**2, 4**) strain; after 1 h (**1, 2**) and 10 h of infection (**3, 4**). Intact bacteria are labeled in green. Bacteria with compromised membranes are labeled with PI and appear in red (or a red spot). Phagosomes are labeled in blue. Scale bars at the bottom right of each panel correspond to 10 µM. (**E**) Quantification of the percentage of dead bacteria. At least 100 bacteria per experiment were scored for PI labeling at 1 h and 10 h post infection. Data are means ± standard deviation from three independent assays.

We used the differential solubilization process previously described [28] to follow the sub-cellular localization of the Δ*gadC* mutant in infected cells (**Figure 3A**). Briefly, the plasma membrane was selectively permeabilized with digitonin. This treatment allowed the detection of cytoplasmic bacteria and proteins. Subsequent treatment with saponin rendered intact phagosomes accessible to antibodies and allowed the detection of intra-phagosomal bacteria. Intracellular localization of the bacteria or LAMP-1 (used as a specific marker of phagosomes) was analyzed using specific antibodies and their co-localization was quantified with the Image J software (**Figure 3B**). Merging of LAMP-1 and bacteria was obtained at all three 3 time-point tested in cells infected with the Δ*gadC* or Δ*FPI* mutant strains. With both mutants, bacterial co-localization with LAMP-1 was elevated (70% and 74%, with Δ*gadC* and Δ*FPI*; respectively) after 1 h and remained very high after 4 h (75% and 64% with Δ*gadC* and Δ*FPI*; respectively) and after 10 h (63% and 64% with Δ*gadC* and Δ*FPI*; respectively). In contrast, co-localization of the wild-type strain with LAMP-1 was only around 24% after 1 h and remained in the same range throughout the infection (20% and 22%, after 4 h and 10 h, respectively). These results strongly suggest that the Δ*gadC* mutant is still trapped in the phagosomal compartment after 10 h, as the Δ*FPI* mutant, whereas the wild-type strain has already escaped into the cytosol after 1 h.

#### Electron microscopy

To confirm this result, we performed thin section electron microscopy of J774.1 cells infected either with wild-type *F. novicida* or with the Δ*gadC* mutant (**Figure 3C**). As expected, significant bacterial replication was observed in the cytosol of most infected cells 10 h post-infection with wild-type *F. novicida* whereas bacterial multiplication was severely impaired in cells infected with the Δ*gadC* mutant. Furthermore, mutant bacteria surrounded by intact phagosomal membrane were still observed after 10 h of infection.

#### Bacterial death

We then determined whether the Δ*gadC* mutant bacteria, trapped in the phagosomal compartment of J774.1 macrophages, remained alive. For this, bacteria were subjected to an intracellular viability assay [29]. As illustrated in **Figure 3D** and quantified (**Figure 3E**), the majority of the replication-deficient Δ*gadC* mutant bacteria (>85%) were alive after 10 h of infection.

Infection of murine bone marrow derived macrophages (BMM) with wild-type *F. novicida* results in activation of the AIM2 inflammasome and pyroptosis [30], [31], [32], which can be monitored by real time incorporation of the membrane-impermeable dye propidium iodide [33]. Therefore, we compared the cell death kinetics of BMM infected either with the wild-type strain or with the Δ*gadC* mutant. As previously shown [33], infection with wild-type *F. novicida* triggered macrophage death after 6–8 h, while infection with the vacuolar mutant (Δ*FPI* mutant) had no effect on cellular viability during the time frame of the experiment. In agreement with its inability to escape from the vacuole and to replicate within host cell, the Δ*gadC* mutant behaved as a Δ*FPI* mutant and was unable to trigger host cell death (**Figure S3**).

### *F. tularensis gadC* encodes a genuine glutamate transporter

**Figure 4:**
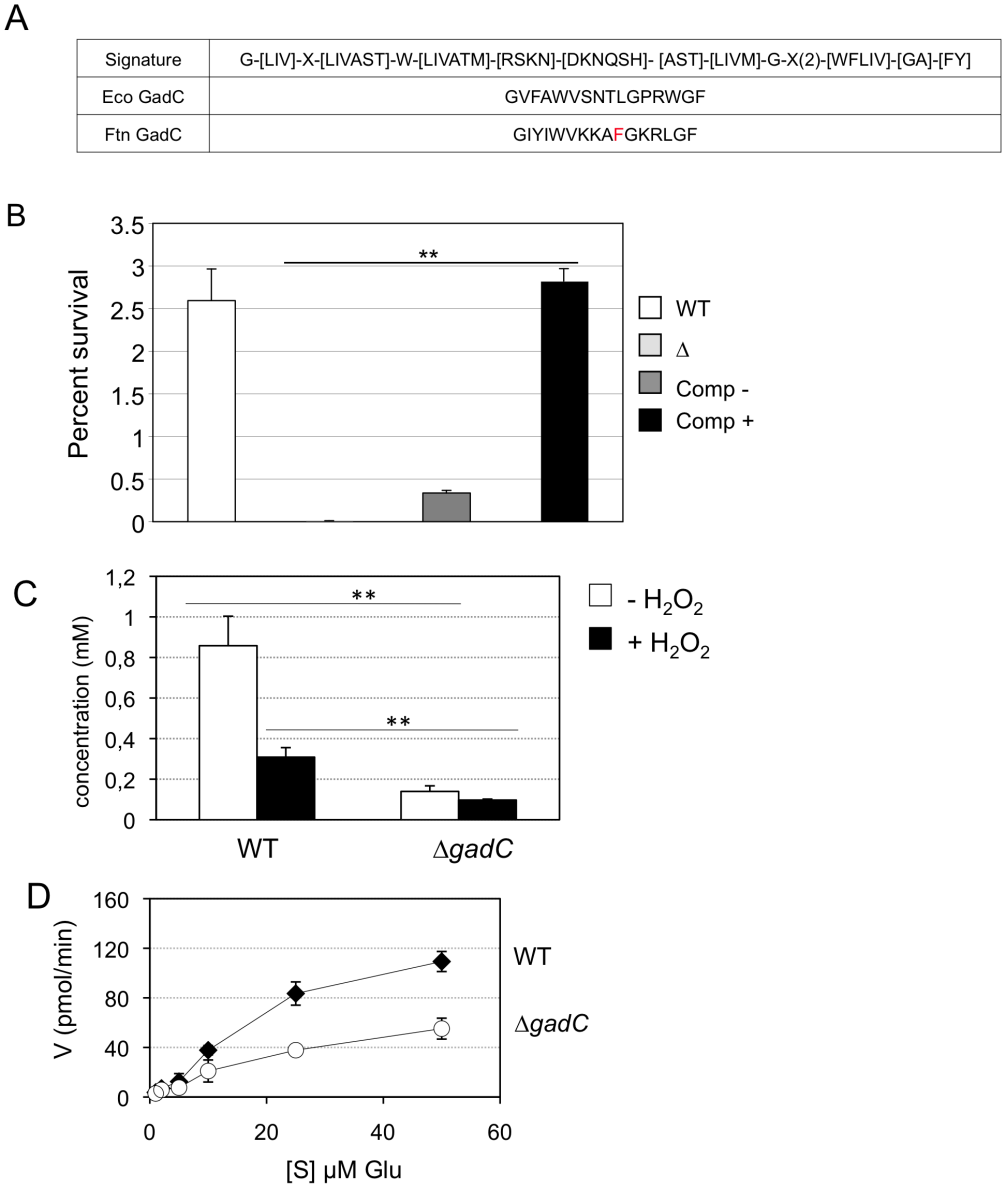
GadC is a glutamic acid transporter. (**A**) The signature sequence for the Glutamate/GABA subfamily of APC transporters is shown in the upper line. Middle line, sequence of the motif present in GadC of *E. coli*; lower line, sequence of the motif present in GadC of *Francisella* (in red the only residue diverging from the consensus). (**B**) Functional complementation of *E. coli gadC*. Acid resistance assays were performed on *E. coli* recombinant strains. Δ: *E. coli* strain bearing an inactivated *gadC* allele. WT: complemented strain bearing the wild-type *E. coli gadC* gene on plasmid pCF348 [49]. Comp −: complemented strain bearing the wild-type *Francisella gadC* gene carried on plasmid pCR2.1-Topo and Comp +: complemented strain bearing the wild-type *Francisella gadC* gene carried on plasmid pCR2.1-Topo and cultivated with IPTG . **p<0.05 as determined by the Student's *t*-test. (**C**) Intracellular glutamate detection and quantification was assayed on exponentially grown bacteria by HPLC analysis. Wild-type *F. novicida* and *ΔgadC* mutant strains were grown in CDM supplemented with 1.5 mM of glutamate, in the absence or presence of H_2_O_2_ (500 µM). **, *p*<0.01 (as determined by the Student's *t*-test). (**D**) Glutamate transport. Kinetics of ^14^C-Glu uptake by wild-type *F. novicida* and Δ*gadC* mutant, at ^14^C-Glu concentrations ranging from 1 µM to 50 µM. Bacteria grown to mid-exponential phase in CDM were tested. Uptake was measured after 5 min incubation with ^14^C-Glu. Ordinate, pmol of glutamate taken up per min (per sample of app. 2.5×10^9^ bacteria). Abscissa, final concentrations of glutamate tested.

Earlier phylogenetic studies have distinguished ten distinct subfamilies within the APC family of transporters, inferring possible substrate specificities. Consensus signature motifs were defined for each of them [34]. Inspection of the *Francisella* GadC protein reveals a signature sequence of the Glutamate-GABA antiporter subfamily in its N-proximal portion (**Figure 4A**), prompting us to test functional complementation of an *E. coli gadC* mutant by the *Francisella gadC* orthologue.

Functional complementation (**Figure 4B**) was determined by comparing the acid resistance (at pH 2.5) of a *gadC*-inactivated strain of *E. coli* (EF491) to the same strain carrying a plasmid-borne *F. novicida gadC* gene (pCRT-*gadC*). As a positive control, we used the *E. coli gadC* mutant complemented with the wild-type *E. coli gadC* gene (EF547). IPTG-induced expression of the *Francisella gadC* allele in the *E. coli gadC* mutant strain restored acid resistance to wild-type level, indicating that the *Francisella* GadC protein displays the acid-resistance function of the *E. coli* GadC protein.

To further support the role of GadC in glutamate entry, we quantified the amounts of intracellular glutamate by HPLC analysis, in the wild-type and *ΔgadC* strains grown in CDM supplemented with 1.5 mM of glutamate (in the presence or in the absence of hydrogen peroxide). As shown in **Fig. 4C**, the concentration of intracellular glutamate was significantly lower in the Δ*gadC* mutant than in the wild-type strain, both in the absence (84% reduction in concentration) or in the presence (31% reduction) of H_2_O_2_. We also quantified the amount of glutamate in culture supernatants of the two strains in the presence of H_2_O_2_ (not shown). External glutamate present in the culture medium of the wild-type strain was 39% lower than that of the Δ*gadC* mutant.

Altogether these data are compatible with a reduced capacity of the Δ*gadC* mutant to take up external glutamate.

We then directly evaluated the impact of *gadC* inactivation on glutamate uptake by live *F. novicida*. For this, we compared the uptake of radiolabeled glutamate (^14^C-Glu) by wild-type *F. novicida* to that of the Δ*gadC* mutant, over a broad range of glutamate concentrations (**Fig. 4D**). Incorporation of ^14^C-Glu was significantly affected in the Δ*gadC* mutant (representing only approximately 50% of the wild-type values at each concentration tested), confirming that GadC is a genuine glutamate transporter. The fact that glutamate uptake was not totally abolished in the Δ*gadC* mutant suggests that other transporter(s) allow the entry of glutamate in this strain.

### The *gadC* mutant shows impaired control of ROS production

**Figure 5:**
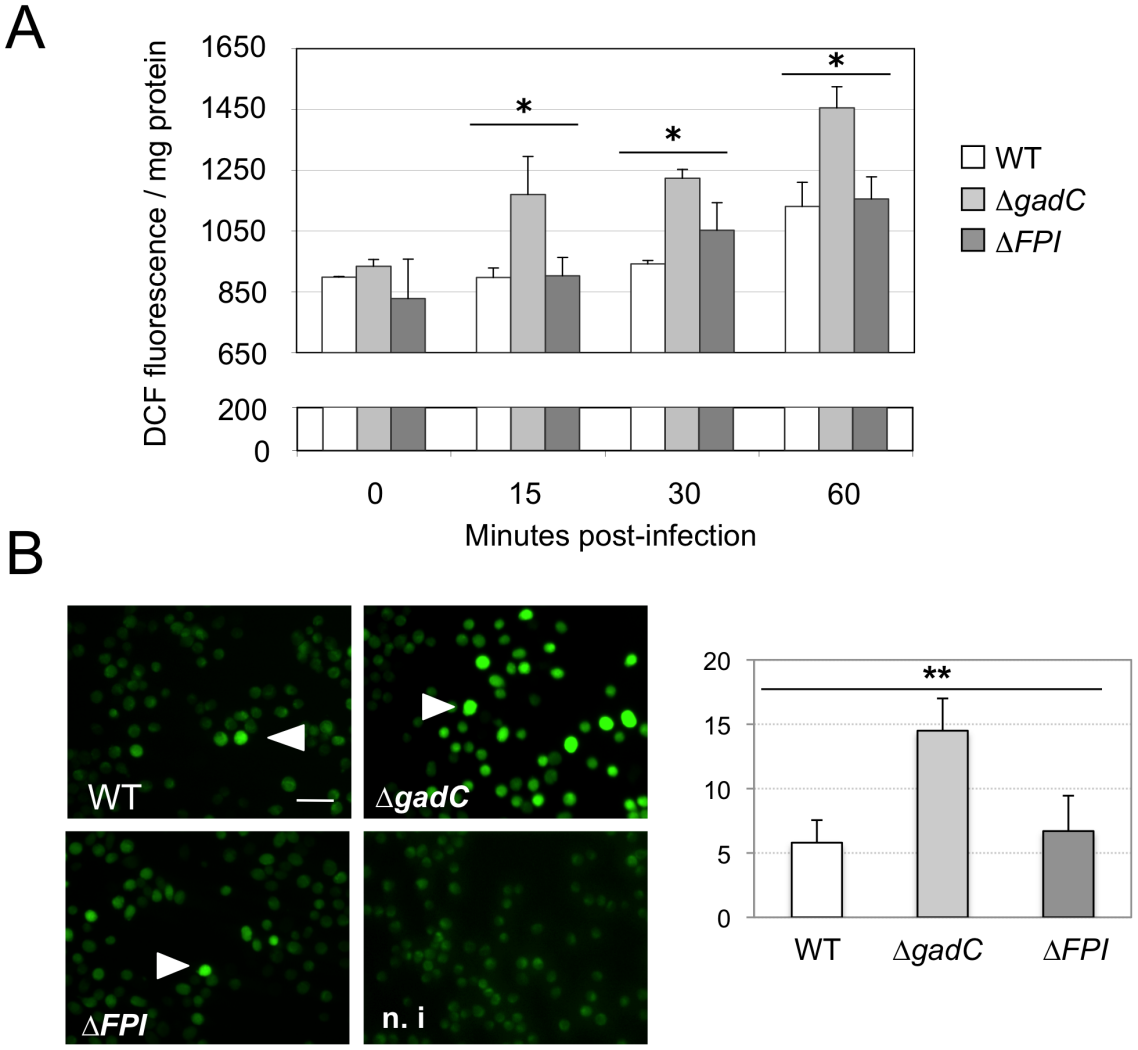
ROS dosage in infected J774.1 cells. (**A**) **ROS dosages.** Generation of ROS was measured by the H_2_DCFDA assay in J774.1 cells infected with wild-type *F. novicida* (WT), the Δ*gadC* or the Δ*FPI* mutant strain. Results, normalized to the protein concentration in each well, are expressed per mg of total protein. The histogram is representative of three independent experiments. (**B**) **Fluorescence microscopy.** Left panel: DCFDA levels were also visualized using fluorescence microscopy. J774.1 cells were infected with wild-type (**1**), Δ*gadC* (**2**) or Δ*FPI* (**3**) bacteria. Non-infected J774.1 cells were used as negative control (**4**). White arrowheads indicate increased DCFDA levels. Scale bar is 50 µm. Images represent fluorescence after 1 h of DCFDA treatment. Typical fields were chosen for illustration. Right panel: Quantification of the percentage of fluorescent J774.1 cells. At least 500 cells per experiment were scored for DCFDA labeling after 1 h of DCFDA treatment. Data are means ± standard deviation from three independent assays.

We compared the amount of reactive oxygen species (ROS) in J774.1 cells infected either with wild-type *F. novicida*, Δ*gadC* or the Δ*FPI* strain, over a 60 min period. For this, we used the H_2_DCF-DA assay (Sigma-Aldrich Co). H_2_DCF-DA is a non-fluorescent cell-permeable compound that has been widely used for the detection of ROS [35]. Once inside the cell, this compound is first cleaved by endogenous esterases to H_2_DCF. The de-esterified product becomes the highly fluorescent compound 2′,7′-dichlorofluorescein (DCF) upon oxidation by ROS. The ROS content increased by 25% after 60 min in cells infected with wild-type *F. novicida* (**Figure 5**). A comparable increase was recorded with the *ΔFPI* mutant. However, in cells infected with the Δ*gadC* mutant, the ROS content was significantly higher than that recorded with the two other strains at each time point (25% higher at 15 min, and 55% higher after 60 min). These results suggest that the Δ*gadC* mutant is affected in its ability to neutralize the production of ROS in the phagosomal compartment. Alternatively, the Δ*gadC* mutant may trigger an increased production of ROS.

### Impaired virulence of the *gadC* mutant is abrogated in NADPH oxidase KO mice

This result prompted us to evaluate the pathogenicity of the Δ*gadC* mutant in mice lacking a functional NADPH oxidase complex, both *in vitro* and *in vivo*.

#### In vitro

**Figure 6:**
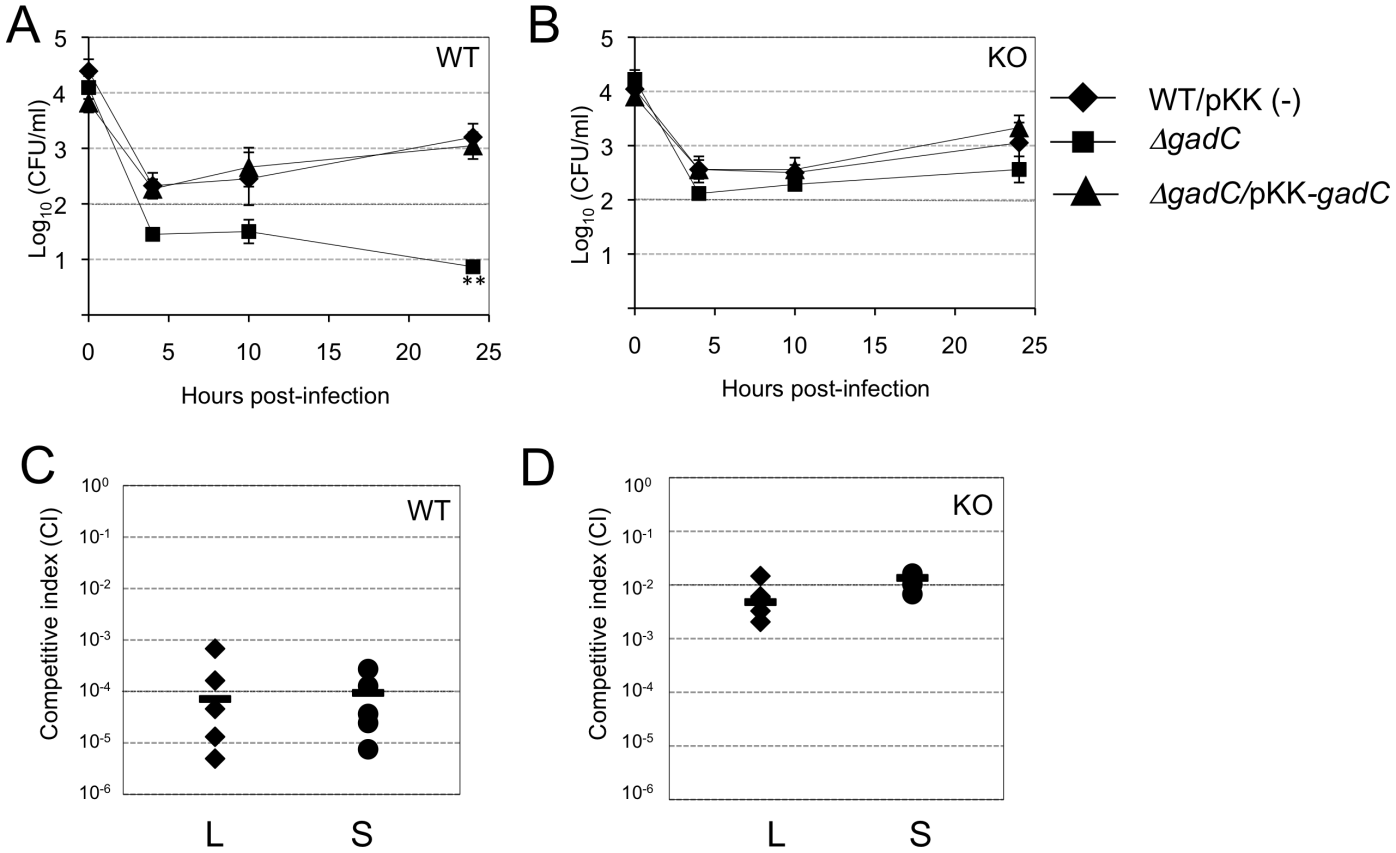
Intracellular survival and virulence in NADPH oxidase KO mice. (**A, B**) Intracellular replication of wild-type *F. novicida* (carrying the empty plasmid pKK214 (WT/pKK(−)), Δ*gadC* mutant and complemented strain (Δ*gadC*/pKK-*gadC*), and Δ*FPI* mutant (Δ*FPI*), was monitored in BMM from either (**A**) C57BL/6J control mice (WT) or (**B**) phox-KO mice (homozygotes gp91*^phox^* ^−/−^; KO), over a 24-h period. Results are shown as the average of log_10_ cfu mL^−1^ ± standard deviation. At all time points tested, the differences between the wild-type and Δ*gadC* mutant values were not statistically different (*p*>0.1, as determined by the Student's *t*-test). (**C, D**) Competition assays were performed by infecting intra-peritoneally: a group of five C57BL/6J control mice (WT, **C**); or a group of five phox-KO mice (KO, **D**), with a 1∶1 mixture of wild-type *F. novicida* and Δ*gadC* mutant strains (100 cfu of each). The data represent the competitive index (CI) value for cfu of mutant/wild-type in the liver (L: black diamonds, left column) and spleen (S: black circles, right column) of each mouse, 48 h after infection. Bars represent the geometric mean CI value.

Intracellular replication of the Δ*gadC* mutant was monitored in bone marrow-derived macrophages from wild-type control mice or homozygotes gp91*^phox^* ^−/−^ in the same C57BL/6J background (designated WT and phox-KO BMMs, respectively) (**Figure 6A, 6B**). Multiplication of wild-type *F. novicida* and the Δ*gadC*/pKK*gadC-*complemented strain was similar at all time points tested in the two types of BMMs. In contrast, the Δ*gadC* mutant showed a significantly more severe intracellular multiplication defect in WT BMMs than in phox-KO BMMs. Indeed, the number of Δ*gadC* mutant bacteria was 10-fold lower than that of wild-type *F. novicida* already after 10 h in WT BMMs, and was 1,000-fold lower after 24 h (**Fig. 6A**). In BMM from phox-KO mice, multiplication of the mutant strain was 1/3 that of wild-type *F. novicida* after 10 h, and 100-fold higher than what was observed in WT macrophages after 24 h (**Figure 6B**). These results showed that multiplication of the Δ*gadC* mutant was mostly restored in BMMs having a defective NADPH oxidase.

#### In vivo

We also performed *in vivo* competition assays in these mice (**Figure 6C, 6D**), as described above for the BALB/c mice. The Competition Index (CI) calculated for both target organs was 10^−4^ in WT mice (**Figure 6C**). In contrast, the CI was 100-fold higher in phox-KO mice (10^−2^, **Figure 6D**). This result comforts the data obtained in BMM cells and demonstrates that, in mice that are unable to produce ROS in the phagosome, the multiplication defect of the Δ*gadC* mutant is partially suppressed. However, the fact that the Δ*gadC* multiplication defect was not completely abolished suggests that oxidative stress may not be the only host restrictive factor.

Altogether, these *in vitro* and *in vivo* data obtained in phox-KO mice strongly suggest that GadC specifically contributes to the ROS defense of *Francisella* in the phagosomal compartment.

### Impact of *gadC* inactivation in glutamate on metabolism

**Figure 7:**
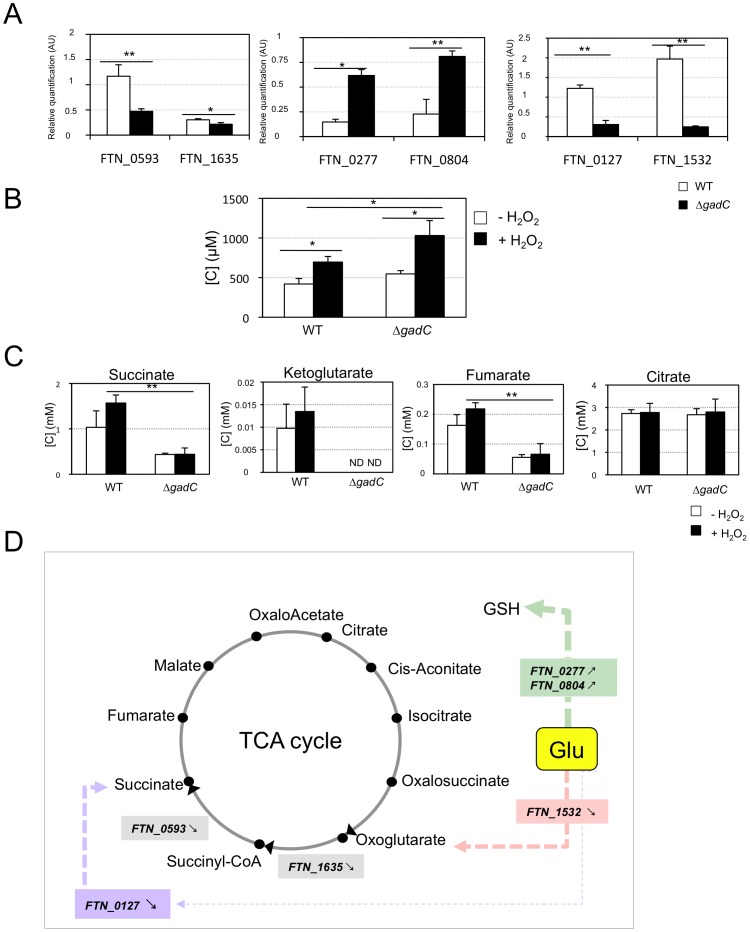
Glutamate transport and metabolism. (**A**) **qRT-PCR of metabolic genes ± H_2_0_2_.** Bacteria were grown in TSB, in the absence or in the presence of H_2_O_2_ (500 µM). qRT-PCR analyses were performed on selected genes, in wild-type *F. novicida* and in the Δ*gadC* mutant. For each gene, the data are represented as the ratios of the value recorded under H_2_O_2_ stress versus non-stress condition. *Left panel:* two genes encoding enzymes of the TCA cycle (*sucA*, *FTN_1635*; *sucD*, *FTN_0593*); m*iddle panel*: two genes encoding enzymes, converting glutamate (Glu) to glutathione (GSH) (*gshA*, *FTN_0277*; *gshB*, *FTN_0804*); r*ight panel:* two genes encoding enzymes, converting Glu to TCA cycle intermediates (*gdhA*, *FTN_1532*; *gabD*, *FTN_0127*). (**B**) **Dosage of glutathione.** The effect of oxidative stress on the cytoplasmic content of glutathione was evaluated in wild-type *F. novicida* and Δ*gadC* mutant strains. Bacteria were cultivated for 30 min, with or without H_2_O_2_ (500 µM), in CDM supplemented with glutamate (1.5 mM). Reduced glutathione was quantified by HPLC analysis. Concentrations [C] are expressed in µM. *p<0.05 as determined by the Student's *t*-test. (**C**) **Dosage of TCA intermediates.** The effect of oxidative stress on the cytoplasmic contents of TCA cycle intermediates was monitored in wild-type *F. novicida* and Δ*gadC* mutant strains. Bacteria were cultivated for 30 min, with or without H_2_O_2_ (500 µM), in CDM supplemented with glutamate (1.5 mM). Succinate, fumarate, citrate and oxoglutarate, were quantified by gas chromatography coupled with mass spectrometry. Concentrations [C] are expressed in mM. *p<0.05, **p<0.01, as determined by the Student's *t*-test. (**D**) **Schematic representation of selected genes involved in glutamate metabolism.** The impact of *gadC* inactivation on the oxidative stress response of the target genes is indicated (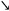 means the ratio (H_2_O_2_-treated/non-treated) is lower in the mutant strain than in the wild-type strain; ↗ the ratio (H_2_O_2_-treated/non-treated) is higher in the mutant strain than in the wild-type strain. In the absence of external glutamate (*e.g.* in standard chemically defined medium), the pool of glutamate present in the bacterial cytoplasm may be synthesized either from oxoglutarate, glutamine, GSH or even proline (according to KEGG metabolic pathways).

Intracellular glutamate plays a central role in a wide range of metabolic processes in bacteria. In order to evaluate the potential impact of the *gadC* inactivation on bacterial glutamate metabolism, we first quantitatively monitored the transcription of selected genes connecting glutamate utilization to either the TCA cycle or to glutathione biogenesis. This analysis was done for wild-type *F. novicida* and for the Δ*gadC* mutant strain, grown in broth with or without H_2_O_2_ (**Figure 7A**).

Expression of *FTN_0593* (*sucD*), *FTN_0127* (*gabD*) and *FTN_1532* (*gdhA*), was significantly decreased in the Δ*gadC* mutant under oxidative stress, whereas their expression was moderately increased in the wild-type strain. Expression of *FTN_*0277(gshA) and *FTN_0804* (*gshB*) was reduced in both strains, under oxidative stress. However, the decrease was significantly less important (app. 4-fold) in the Δ*gadC* mutant than in the wild-type strain. Expression of *FTN_1635* (*sucA*) was significantly decreased in both strains under oxidative stress. These data indicate that the absence of *gadC* affects the expression of several genes linked to glutamate metabolism under oxidative stress. The fact that expression of the *gadC* gene itself was significantly upregulated (approximately 10-fold) in the wild-type strain exposed to H_2_O_2_ stress (not shown) supports the importance of the GadC transporter in oxidative stress defense.

Direct quantification of TCA cycle intermediates present in the cytoplasm of the wild-type and Δ*gadC* strains, by gas chromatography coupled with mass spectrometry (see Materials and methods for details), revealed that *gadC* inactivation significantly affected succinate, fumarate, and oxoglutarate contents (**Figure 7C**). Indeed, in the Δ*gadC* mutant, the concentrations of succinate and fumarate were reduced *ca.* 60% as compared to the wild-type strain, whereas oxoglutarate was below the detection threshold of the assay. The concentrations of the three molecules increased up to 40% in the wild-type strain exposed to oxidative stress, suggesting an activation of the TCA under this condition. The concentrations of succinate and fumarate were not significantly modified in the Δ*gadC* mutant upon oxidative stress and oxoglutarate production was still below detection. The concentration of citrate was similar in the wild-type and the Δ*gadC* mutant and did not vary upon oxidative stress, in any of the two strains. The intracellular concentrations of glutathione were also almost similar in the wild-type and Δ*gadC* mutant (**Figure 7B**). Remarkably, under oxidative stress, the intracellular concentration of glutathione increased in both strains but only reached 65% of the level of the Δ*gadC* mutant in the wild-type strain.

These observations prompted us to evaluate the impact of supplementation with different TCA cycle intermediates on survival of the Δ*gadC* mutant in response to H_2_O_2_ challenge. For this, exponential phase wild-type and *ΔgadC* mutant strains, diluted in CDM supplemented with glutamate, were subjected to oxidative stress, in the presence or absence of either fumarate, succinate or oxoglutarate (**Figure S5**). The sensitivity to H_2_O_2_ of the *ΔgadC* mutant was not modified neither by fumarate nor by oxoglutarate. In contrast, supplementation with succinate increased significantly the survival of the *ΔgadC* mutant, to nearly wild-type level.

## Discussion

Intracellular pathogenic bacteria have adapted a variety of strategies and specific intracellular niches for survival and multiplication within their host [36]. Some reside in a vacuolar compartment whereas others have evolved to gain access to the host cytosol for multiplication. In mammalian host cells, *Francisella* intracellular replication occurs exclusively in the cytosolic compartment. We show here that inactivation of the GadC permease of *Francisella* prevents phagosomal escape, thus severely altering bacterial intracellular multiplication and virulence.

The data presented suggest that the GadC protein of *Francisella* is required to resist to the oxidative burst triggered by the NADPH oxidase in the phagosomal compartment of infected macrophages. We propose that GadC-mediated entry of glutamate contributes to fuel the tricarboxylic acid cycle and modulates the redox status of the bacterium. This work thus provides insights into the possible links between oxidative stress resistance, metabolism, and bacterial intracellular parasitism.

### GadC is involved in oxidative stress defense and required for phagosomal escape

Inactivation of *gadC* in *F. novicida* led to a severe growth defect in all cell types tested and *in vivo* assays further demonstrated the importance of GadC in *Francisella* virulence. Confocal and electron microscopy analyses revealed that the severe intracellular growth defect of the mutant was due to its inability to escape from the phagosomal compartment of infected macrophages.

Interestingly, most of the mutant bacteria that remained trapped within the phagosome were still alive for at least 10 h post-infection, indicating that impaired escape was not due to bacterial death. Since the Δ*gadC* mutant showed increased susceptibility to oxidative stress in broth and failed to efficiently neutralize reactive oxygen species production in cells, it is likely that ROS may predominantly affect bacterial escape rather than survival.

*F. tularensis* produces enzymes that can metabolize and neutralize ROS, such as a superoxide dismutases (SodB, SodC), a catalase (KatG), a glutathione peroxidase and a peroxireductase [9], [10]. Acid phosphatases have also been implicated in the resistance of intracellular *Francisella* to H_2_O_2_ generated in the phagosomal compartment by the NADPH oxidase ([37], [38] and references therein). However, inactivation of the major phosphatase *acpA* in *F. tularensis* subsp. *tularensis*, had no impact on the activity of the NADPH oxidase in human neutrophils [5], thus confirming that other *Francisella* factors were involved in NADPH oxidase inhibition.

We show here that *Francisella* GadC is an important player specifically involved in oxidative stress defense. The existence of several paralogues of both the transporter GadC and the decarboxylase GadB in some bacterial species (for example in *L. monocytogenes*) might account for the fact that these have not yet been found to contribute to oxidative stress resistance and intracellular survival in standard genetic screens. Indeed, if functional paralogues exist, they must be simultaneously inactivated to observe a possible phenotypic defect. In addition, isofunctional antiporters with no significant amino acid sequence similarity to the GadC protein might exist in these bacteria.

The contribution of the GAD system to intracellular survival critically depends on the cellular compartment where bacterial survive and multiply. Indeed, bacteria residing in vacuolar compartments (such as *Salmonella, Mycobacteria, Legionella, Brucella and Chlamydia*) encounter different types of stresses (pH, oxidative, nutritional,…) than bacteria able to multiply in the host cell cytosol (such as *Francisella*, *Listeria*, *Shigella* and *Rickettsia*).

L-glutamate is very abundant in the intracellular compartment (reported concentrations vary between 2 and 20 mM) when compared to the extracellular compartment (app. 20 µM) [39]. Human macrophages have both the cystine/glutamate transporter and the Na-dependent high-affinity glutamate transporters (excitatory amino acid transporters, EAATs) that transport glutamate and aspartate. To maintain their intracellular pool of glutamate, macrophages may use either these transporters to import glutamate from the extracellular milieu or enzymatically convert cytosolic glutamine (via glutaminase) and aspartate (via aspartate transpeptidase) to glutamate. Glutamate might also be produced spontaneously intracellularly from pyroglutamate. Currently, nothing is known with respect to the content of glutamate in the phagosomal compartment. This might prove extremely difficult to establish, especially for pathogens such as *Francisella* or *Listeria* that reside only very transiently in this compartment.

A limited number of bacterial species have been shown to possess a GAD system [40]. These include *E. coli*, *Lactobacillus*, *L. monocytogenes* and *Shigella* species, in which the GAD system plays a major role in acid tolerance. It has been suggested that the GAD system is important for pathogenic microorganisms that, upon oral infection of mammalian hosts, need to survive the low pH of the stomach. However, some enteric pathogens like *Salmonella* do not possess a function GAD system and must thus rely on other anti-acidic pH strategies. Interestingly, the GAD system has been also found to contribute to oxidative stress defense in yeast and plant [41]. In bacteria, molecules such as the NADPH and NADH pools and glutathione (GSH), contribute to oxidative stress defense. Reduced GSH, present at mM concentrations, maintain a strong reducing environment in the cell. Specific enzymes are also dedicated to control the levels of reactive oxygen species (ROS).

Remarkably, the Δ*gadC* mutant was still outcompeted by wild-type bacteria in phox-KO mice. The different environments and the immune pressure, encountered by the bacterium during its systemic dissemination, are probably far more complex than in culture systems. *In vivo*, *Francisella* GadC is thus likely to contribute to other functions than combat ROS in the phagosomal compartment. It may, for instance, fulfill classical nutritional functions during bacterial cytosolic multiplication (in macrophages and/or in other infected non-phagocytic cells). Alternatively, GadC may be required during the bacterial blood stage multiplication and dissemination of the bacterium.

### A link between oxidative stress and bacterial metabolism

In *E. coli*, GABA produced by the glutamate decarboxylase is metabolized via the GABA shunt pathway. This leads to the production of succinate via the consecutive action of two enzymes: a GABA/oxoglutarate amino-transferase (GabT) that removes the amino group from GABA to form succinic semialdehyde (SSA) and Glu; and a succinic semialdehyde dehydrogenase (GabD) that oxidises SSA to form succinate. Very recently, Karatzas and co-workers have shown [42] that *L. monocytogenes* also possessed functional GabT and GabD homologues that could provide a possible route for succinate biosynthesis in *L. monocytogenes*. The GABA shunt pathway, allowing the bypass of two enzymatic steps of the TCA (from oxoglutarate to succinate; **Figure 7** **and S6**), is thought to play a role in glutamate metabolism, anaplerosis and antioxidant defense. However, its physiological role in pathogenesis is yet poorly understood. *Francisella* genomes possess a *gabD* orthologue but lack *gabT*. The GABA shunt pathway may therefore be non-functional in *Francisella*. Interestingly, the isogenic glutamate decarboxylase Δ*gadB* mutant (**Figure S4**) that we constructed, showed a much less severe intracellular multiplication defect than the Δ*gadC* mutant, and as well as no (or only a very mild) attenuation of virulence. If the glutamate imported via GadC would serve to produce GadB-mediated GABA, one would expect *gadB* inactivation to cause the same defect as *gadC* inactivation. As already mentioned in the Introduction, the *gadB* orthologue encodes a truncated protein in the subspecies *holarctica*. Altogether, these data support the notion that GadC and GadB of *Francisella* do not function in concert, unlike in several other bacterial species, and that GABA production plays a marginal role in *Francisella* pathogenesis. Further work will be required to understand the exact contribution of GadB in *Francisella* metabolism.

Our data indicate that GadC of *Francisella* encodes a genuine glutamate transporter involved in oxidative stress, unlike most other GadC orthologues described thus far. Glutamate can be converted in the bacterial cytoplasm into a number of compounds (**Figure 7**), such as glutamine, glutathione, GABA or the TCA cycle intermediate oxoglutarate. Oxoglutarate is known to be a potent anti-oxidant molecule that can be converted, in absence of any enzymatic reaction, into succinate in the presence of H_2_O_2_. In addition, conversion of glutamate to oxoglutarate by the glutamate dehydrogenase GdhA increases the production of NADPH, which might also contribute to the anti-oxidant effect of glutamate acquisition.

Quantitative analyses of the intra-bacterial content of TCA cycle intermediates (**Figure 7B**) revealed a significant reduction of succinate and fumarate in the *gadC* mutant, as compared to wild-type *F. novicida*, and a striking decrease of oxoglutarate. These data support the notion that reduced entry of glutamate directly affects the production of these TCA cycle intermediates. In contrast, the amount of citrate remained unchanged in the mutant, suggesting refueling of the TCA cycle via other entry points (such as glycolysis or amino acid conversion).

Of note, mutants in genes *gdhA* (*FTN_1533*) and *gabD* (*FTN_0127*) were identified as required for replication in *D. melanogaster* S2 cells in a recent screen, supporting a role for these genes in intracellular bacterial survival [43]. The production and utilization of oxoglutarate by *Francisella* may thus constitute an efficient mean to modulate its cytoplasmic concentration of ROS.

In the absence of external glutamate, the pool of intracellular glutamate may be synthesized either from oxoglutarate, glutamine, GSH or even proline (according to KEGG metabolic pathways). Therefore, we evaluated the impact of *gadC* inactivation on the expression of genes involved in glutamate metabolism, under oxidative stress conditions. qRT-PCR analyses were performed in wild-type *F. novicida* and in the Δ*gadC* mutant, grown in chemically defined medium containing glutamate, in the absence or in the presence of H_2_O_2_ (**Figure 7A**). These assays revealed that *gadC* inactivation led to an important down-regulation of the genes involved the conversion of glutamate to oxoglutarate and succinate, upon oxidative stress (*FTN_1532* and *FTN_0127*, respectively). Conversely, *gadC* inactivation only moderately decreased the expression of *gshA* and *gshB*, the two genes involved in glutathione biosynthesis (*FTN_0277* and *FTN_0804*), upon oxidative stress whereas the expression of these genes was severely decreased in the wild-type strain.

These data are compatible with the notion that, under oxidative stress, the wild-type strain may favor the conversion of a fraction of its cytoplasmic pool of glutamate (neosynthesized and imported) to produce oxoglutarate and succinate rather than GSH. In contrast, when the cytosolic pool of glutamate is restricted to neosynthesized glutamate (*i.e.* in a *gadC* mutant or in a glutamate-depleted medium), the production of oxaloglutarate and succinate may be decreased to favor that of other molecules (including GSH).

In conclusion, we identified a glutamate transporter as a novel *Francisella* virulence attribute that suggests links between the oxidative stress response and the TCA cycle during the early stage of the bacterial intracellular life cycle. The importance of the TCA cycle in the homeostasis of reactive oxygen species has just started to be considered in pathogenic bacterial species [12], [44], [45], [46]. The development of specific inhibitors of transport systems involved in intracellular adaptation might constitute interesting anti-bacterial therapeutic targets.

## Materials and Methods

### Ethics statement

All experimental procedures involving animals were conducted in accordance with guidelines established by the French and European regulations for the care and use of laboratory animals (Decree 87–848, 2001–464, 2001–486 and 2001–131 and European Directive 2010/63/UE) and approved by the INSERM Ethics Committee (Authorization Number: 75-906).

### Bacterial strains, media, and chemicals

*F. tularensis* subsp. *novicida* (*F. novicida*) strain U112, its Δ*FPI* derivative, and all the mutant strains constructed in this work, were grown as described in **Supplementary Material**. *E. coli* strains (kindly provided by John Foster, University of South Alabama, USA) were grown as described in **Supplementary Material**. All bacterial strains, plasmids, and primers used in this study are listed in **Supplemental Table 1**.

Details of the construction and characterization of mutant and complemented strains; macrophage preparation and infections, are described in **Supplementary Material**. Quantitative (q)RT-PCR (real-time PCR) was performed with gene-specific primers (**Supplemental Table 1**), using an ABI PRISM 7700 and SYBR green PCR master mix (Applied Biosystems, Foster city, CA, USA).

Electron and confocal microscopy complete descriptions; real time cell death and phagosome permeabilization assays, are described in **Supplementary Material**.

### Multiplication in macrophages

J774.1 macrophage-like cells (ATCC Number: TIB-67) were propagated in Dulbecco's Modified Eagle's Medium (DMEM) containing 10% fetal calf serum, whereas human monocyte-like cell line THP-1 (ATCC Number: TIB-202) and bone marrow-derived macrophages (BMM) from BALB/c were propagated in RPMI Medium 1640 containing 10% fetal calf serum, respectively. J774.1 and BMM were seeded at a concentration of ∼2×10^5^ cells per well in 12-well cell tissue plates and monolayers were used 24 h after seeding. THP-1 were seeded at a concentration of ∼2×10^5^ cells per well in 12-well cell tissue plates 48 h before infection, and supplemented with phorbol myristate acetate (PMA) to induce cell differentiation (200 ng/ml). J774.1, BMM and THP-1 were incubated for 60 min at 37°C with the bacterial suspensions (approximately multiplicities of infection 100) to allow the bacteria to enter. After washing (time zero of the kinetic analysis), the cells were incubated in fresh culture medium containing gentamicin (10 µg mL^−1^) to kill extracellular bacteria. At several time-points, cells were washed three times in DMEM or RPMI, macrophages were lysed by addition of water and the titer of viable bacteria released from the cells was determined by spreading preparations on Chocolate agar plates. For each strain and time in an experiment, the assay was performed in triplicate. Each experiment was independently repeated at least three times and the data presented originate from one typical experiment.

### Isolation of total RNA and reverse transcription

Bacteria were centrifuged for 2 min in a microcentrifuge at room temperature and the pellet was quickly re-suspended in Trizol solution (Invitrogen, Carlsbad, CA, USA). Samples were either processed immediately or frozen and stored at −80°C. Samples were treated with chloroform and the aqueous phase was used in the RNeasy Clean-up protocol (Qiagen, Valencia, CA, USA) with an on-column DNase digestion of 30 min [47].

RNA Reverse transcription (RT)-PCR experiments were carried out with 500 ng of RNA and 2 pmol of specific reverse primers. After denaturation at 65°C for 5 min, 6 µL of the mixture containing 4 µL of 5× first strand buffer and 2 µL of 0,1 M DTT were added. Samples were incubated 2 min at 42°C and, then, 1 µL of Superscript II RT (Thermo Scientific) was added. Samples were incubated for 50 min at 42°C, heated at 70°C for 15 min and chilled on ice. Samples were diluted with 180 µL of H_2_O and stored at −20°C.

The following pair of primers was used to amplify the mRNA corresponding to the transcript of *FTN_0570* (p13/p14), *FTN_0571* (p15/p16), *FTN_1700* (p27/9p28), *FTN_1701* (p29/p30), *FTN_1702* (p31/p32), *FTN_1532* (p33/p34), *FTN_0127* (p35/p36), *FTN_0277* (p37/p38), *FTN_0804* (p39/p40), *FTN_0593* (p41/p42), *FTN_1434* (p43/p44) and *FTN_1635* (p45/p46) (**Supplemental Table 1**).

### Quantitative real-time RT-PCR

Wild-type *F. novicida* and mutant strains were grown at 37°C from OD_600_ ∼0.1. After 4 h of incubation, samples were harvested and RNA was isolated. For oxidative stress tests, samples were cultivated 30 min more with or without H_2_O_2_ (500 µM). The 25 µL reaction consisted of 5 µL of cDNA template, 12.5 µL of Fastart SYBR Green Master (Roche Diagnostics), 2 µL of 10 µM of each primer and 3.5 µL of water. qRT-PCR was performed according manufacturer's protocol on Applied Biosystems - ABI PRISM 7700 instrument (Applied Biosystems, Foster City, CA). To calculate the amount of gene-specific transcript, a standard curve was plotted for each primer set using a series of diluted genomic DNA from wild-type *F. novicida*. The amounts of each transcript were normalized to helicase rates (*FTN_1594*).

### Oxidative and pH stress survival assays

Stationary-phase bacterial cultures were diluted at a final OD_600_ of 0.1 in TSB broth or CDM with or without glutamate (1.5 mM final). Exponential-phase bacterial cultures were diluted to a final concentration of 10^8^ bacteria mL^−1^ and subjected to either 500 µM H_2_O_2_ or pH 5.5.

Oxidative stress response was also tested in CDM supplemented with glutamate, in the presence or absence of the TCA cycle intermediates: oxoglutarate, succinate or fumarate (1.5 mM final). The number of viable bacteria was determined by plating appropriate dilutions of bacterial cultures on Chocolate Polyvitex plates at the start of the experiment and after the indicated durations. Cultures (5 mL) were incubated at 37°C with rotation (100 rpm) and aliquots were removed at indicated times, serially diluted and plated immediately. Bacteria were enumerated after 48 h incubation at 37°C. Experiments were repeated independently at least twice and data represent the average of all experiments.

### Confocal experiments

J774.1 cells were infected with wild-type *F. novicida*, *ΔgadC* or *ΔFPI* strains for 1 h, 4 h and 10 h at 37°C, and were washed in KHM (110 mM potassium acetate, 20 mM Hepes, 2 mM MgCl_2_). Cells were incubated for 1 min with digitonin (50 µg/mL) to permeabilize membranes. Then cells were incubated for 15 min at 37°C with primary anti *F. novicida* mouse monoclonal antibody (1/500 final dilution, Immunoprecise). After washing, cells were incubated for 15 min at 37°C with secondary antibody (Ab) (Alexa Fluor 488-labeled GAM, 1/400 final dilution, Abcam) in the dark. After washing, cells were fixed with PFA 4% for 15 min at room temperature (RT) and incubated for 10 min at RT with 50 mM NH_4_Cl to quench residual aldehydes. After washing with PBS, cells were incubated for 30 min at RT with primary anti-LAMP1 Ab (1/100 final dilution, Abcam) in a mix with PBS, 0.1% saponine and 5% goat serum. After washing with PBS, cells were incubated for 30 min at RT with secondary anti-rabbit Ab (alexa 546-labeled, 1/400 dilution, Abcam). DAPI was added (1/5,000 final dilution) for 1 min. After washing, the glass coverslips were mounted in Mowiol. Cells were examined using an X63 oil-immersion objective on a LeicaTSP SP5 confocal microscope. Co-localization tests were quantified by using Image J software; and mean numbers were calculated on more then 500 cells for each condition. Confocal microscopy analyses were performed at the Cell Imaging Facility (Faculté de Médecine Necker Enfants-Malades).

### Electron microscopy

Infection of J774.1 cells was followed by thin section electron microscopy as previously described [48].

### Determination of intracellular bacterial viability

To evaluate the viability of *F. tularensis*, labelings were adapted to use the cell-impermeant nucleic acid dye propidium iodide (PI). J774.1 macrophage-like cells were seeded at 5.10^5^cells/ml on glass coverslips in 12-well bottom flat plates. Next day, cells were infected for 10 h with wild-type *F. novicida* or *ΔgadC* strain. After infection, cells were first permeabilized with digitonin for 1 min, washed three times with KHM and incubated for 12 min at 37°C with 2.6 µM PI (Life technologies, L7007) in KHM buffer to label compromised bacteria in permeabilized cells. Cells were washed three times with KHM and incubated for 15 min at 37°C with primary anti *F. novicida* mouse monoclonal antibody (1/500 final dilution). After washing, cells were incubated for 15 min at 37°C with secondary antibody (Ab) (Alexa Fluor 488-labeled GAM, 1/400 final dilution) in the dark. After washing, cells were fixed with PFA 4% for 15 min at room temperature (RT) and incubated for 10 min at RT with 50 mM NH_4_Cl to quench residual aldehydes. After washing with PBS, cells were incubated for 30 min at RT with primary anti-LAMP1 Ab (1/100 final dilution) in a mix with PBS, 0.1% saponine and 5% goat serum. After washing with PBS, cells were incubated for 30 min at RT with secondary anti-rabbit Ab (alexa 405-labeled, 1/400 dilution). After washing, the glass coverslips were mounted in Mowiol. Cells were examined using an X63 oil-immersion objective on a LeicaTSP SP5 confocal microscope. Analysis of cell fluorescence was performed with Image J software (http://rsb.info.nih.gov/ij).

### ROS detection assay

Intracellular reactive oxygen species (ROS) were detected by using the oxidation-sensitive fluorescent probe dye, 2′,7′-dichlorodihydrofluorescein diacetate (H_2_DCF-DA) as recommended by the manufacturer (CM-H2DCF-DA, Molecular Probes, Eugene, OR). J774.1 cells were seeded at 5.10^4^ cells/well. Cells were infected with bacteria for 15 min (MOI of 100∶1), washed three times with PBS and incubated with H_2_DCF-DA diluted in PBS (concentration). DCF fluorescence was measured with the Victor^2^ D fluorometer (Perkin-Elmer, Norwal, CT) with the use of excitation and emission wavelengths of 480 nm and 525 nm, respectively. Values were normalized by protein concentration in each well (Bradford). Samples were tested in triplicates in three experiments.

### Determination of ROS generation via fluorescent microscopy

J774.1 cells were seeded at 5.10^4^ cells/well. Cells were infected with bacteria for 15 min (MOI of 1,000∶1), washed three times with PBS and incubated with H_2_DCF-DA diluted in PBS for 1 h (5 µM). Images of the cells were captured with an Olympus CKX41 microscope and treated with Image J software. Cell counts were performed over 10 images of approximately 50 cells.

### Acid resistance assay

Acid resistance tests in *E. coli* were performed at pH 2.5 as described previously [49], by comparing the number of survival treated cells after 1 h of treatment over the number of cells at T0. We compared survival of wild-type *E. coli* strain (WT) with *E. coliΔgadC* (Δ) and *E. coliΔgadC* complemented with the *F. novicida gadC* gene (PCR-amplified gene *FTN-0571* introduced into plasmid pCR2.1-Topo, in the correct orientation downstream of the p*lac* promoter) (Comp). Acid challenge was performed by diluting 1∶100 the overnight (22 h) culture in LB, supplemented (Comp +) or not (Comp −) with 10^−4^ M final IPTG.

### Glutamate assays

Glutamate detection and quantification was done by using HPLC analysis. Wild-type *F. novicida* and *ΔgadC* strains were tested in CDM supplemented with 1.5 mM of glutamate, with or without H_2_O_2_ (500 µM). For each condition, three independent cultures were prepared by overnight growth in CDM. The overnight cultures were diluted with 50 mL of fresh medium to OD_600_ of 0.05 and cultivated to an OD_600_ of app. 0.35. Bacteria were harvested by centrifugation at 4,000× *g* for 20 min, resuspended in 25 mL of pre-warmed appropriated medium and cultivated for 30 min. For extracellular glutamate dosage, 100 µL of each supernatant were resuspended with 400 µL of cold methanol and centrifuged at 12,000× *g* for 5 min. 20 µL of each preparation were derivatized with 80 µL of OPA. For intracellular dosage, each sample were resuspended in 600 µL of cold methanol. The bacterial suspensions were sonicated thrice for 30 sec at 4.0 output, 70% pulsed (Branson Sonifier 250). Lysates were then centrifuged at 3,300× *g* for 8 min, to remove debris.

Following steps were done with the standard procedure of Agilent using ZORBAX Eclipse AAA high as HPLC column. An amount equivalent to 2 µL of each sample was injected on a Zorbax Eclipse-AAA column, 5 µm, 150×4.6 mm (Agilent), at 40°C, with fluorescence detection. Aqueous mobile phase was 40 mM NaH_2_PO_4_, adjusted to pH 7.8 with NaOH, while organic mobile phase was acetonitrile/methanol/water (45/45/10 v/v/v). The separation was obtained at a flow rate of 2 mL min^−1^ with a gradient program that allowed for 2 min at 0% B followed by a 16-min step that raised eluent B to 60%. Then washing at 100% B and equilibration at 0% B was performed in a total analysis time of 38 min. To evaluate glutamate concentration, glutamate standard curve was made in parallel.

### Glutathione assays

The procedure for the measurement of GSH was previously described [50]. Briefly, GSH were separated by HPLC, equipped with a Shimadzu Prominence solvent delivery system (Shimadzu Corp., Kyoto, Japan), using a reverse-phase C18 Kromasil (5 µm; 4.6×250 mm), obtained from AIT (Paris, Fr). The mobile phase for isocratic elution consisted of 25 mmol L^−1^ monobasic sodium phosphate, 0.3 mmol L^−1^ of the ion-pairing agent 1-octane sulfonic acid, 4% (v/v) acetonitrile, pH 2.7, adjusted with 85% phosphoric acid. The flow rate was 1 mL min^−1^. Under these conditions, the separation of aminothiol was completed in 20 min. Deproteinated samples were injected directly onto the column using a Shimadzu autosampler (Shimadzu Corp.). Following HPLC separation, GSH was detected with a model 2465 electrochemical detector (Waters, MA, USA) equipped with a 2 mm Glassy carbon (GC) analytical cell and potential of +700 mV were applied.

### Glutamate transport assays

Cells were grown in Chamberlain medium to mid-exponential phase and then harvested by centrifugation and washed twice with Chamberlain without amino acid. The cells were suspended at a final OD_600_ of 0.5 in the same medium containing 50 mg/ml of chloramphenicol. After 15 min of pre-incubation at 25°C, uptake was started by the addition of L-[U-^14^C] glutamic acid (Perkin Elmer), at various concentrations (^14^C-Glu ranging from 1 to 50 µM). The radiolabeled ^14^C-Glu was at a specific activity of 9.25 GBq.mmol ^−1^. Samples (100 µL of bacterial suspension) were removed after 5 min and collected by vacuum filtration on membrane filters (Millipore type HA, 25 mm, 0.22 mm) and rapidly washed with Chamberlain without amino acid (2×5 mL). The filters were transferred to scintillation vials and counted in a Hidex 300 scintillation counter. The counts per minute (c.p.m.) were converted to picomoles of amino acid taken up per sample, using a standard derived by counting a known quantity of the same isotope under similar conditions.

### Quantification of TCA cycle intermediates

Succinate, fumarate, citrate and oxoglutarate were quantified by gas chromatography coupled with mass spectrometry (GC/MS). Wild-type *F. novicida* and Δ*gadC* strains were grown as for glutamate quantification. Briefly, after an overnight preculture in CDM, three independent cultures of wild-type and Δ*gadC* mutant were cultivated in 50 mL CDM to an OD_600_ of app. 0.35. Bacteria were harvested by centrifugation at 4,000× g for 15 min, resuspended to the same OD_600_ in pre-warmed CDM supplemented with glutamate (1.5 mM) and cultivated for 30 min±500 µM H_2_O_2_.

Metabolite measurements were normalized by checking that each sample contained equal amounts of total proteins.

#### Protein dosage

After centrifugation at 4,000× g for 20 min, each sample was resuspended in 2 mL of ice-cold 5 mM Tris.HCl (pH8). The bacterial suspensions were sonicated thrice for 30 sec at 4.0 output, 70% pulsed (Branson Sonifier 25). Lysates (three per strain per condition) were then centrifuged at 3,300× *g* for 8 min, to remove debris. Protein concentration of the different samples was determined by the BCA assay (Pierce), following the manufacturer's recommendation. The values recorded for the wild-type and the *ΔgadC* mutant, in the absence or in the presence of H_2_O_2_, were not significantly different (1,000 mg mL^−1^±10 mg mL^−1^; *p* value≥0.5). The assay was repeated twice.

#### Metabolite measurements

After oxidative stress, bacterial samples were centrifugated at 4,000× g for 20 min, resuspended in 600 µl of cold methanol. The bacterial suspensions were sonicated thrice for 30 sec at 4.0 output, 70% pulsed (Branson Sonifier 250). Lysates were then centrifuged at 3,300× *g* for 8 min, to remove debris. Then, samples were purified through SPE column (Strata-X, 30 mg mL^−1^, Phenomenex, California, USA). After elution, complete lyophilization and derivatization with methoxymation and syliation steps, 1 µl of each sample were injected on GC column coupled to detection mass. Analyses were performed on Shimadzu GC2/MS-2010 (Columbia, MD). Data represent the average of three independent cultures for each condition.

### Virulence determination

Wild-type *F. novicida* and Δ*gadC* mutant strains were grown in TSB to exponential growth phase and diluted to the appropriate concentrations. 6 to 8-week-old female BALB/c mice (Janvier, Le Genest St Isle, France) were intra-peritoneally (i.p.) inoculated with 200 µl of bacterial suspension. The actual number of viable bacteria in the inoculum was determined by plating appropriate dilutions of bacterial cultures on Chocolate Polyvitex plates. For competitive infections, wild-type *F. novicida* and mutant bacteria were mixed in 1∶1 ratio and a total of 100 bacteria were used for infection of each of five mice. After two days, mice were sacrificed. Homogenized spleen and liver tissue from the five mice in one experiment were mixed, diluted and spread on to chocolate agar plates. Kanamycin selection to distinguish wild-type and mutant bacteria were performed. Competitive index (CI) [(mutant output/WT output)/(mutant input/WT input)]. Statistical analysis for CI experiments was as described in [51]. Macrophage experiments were analyzed by using the Student's *t*-test.

## Supporting Information

Figure S1 **The** ***gadC*** **region.** (**A**) **Schematic organization.** The gene *gadC* (*FTN_0571*, grey arrow) is flanked, upstream (83 bp) by gene *FTN_0572* (transcribed on the opposite strand); and downstream, by gene *FTN_0570* (white arrows), separated by a 101 bp intergenic region. (**B**) **Transcriptional analysis.** We performed rapid amplification of cDNA ends (5′-RACE) to determine the 5′ end of the *gadC* mRNA. A broken arrow shows the transcription start of *gadC* (+1). Inspection of the sequence immediately upstream of the transcriptional start identified putative -10 and -35 promoter elements that share homology to the consensus site recognized by the major sigma factor σ^70^ [52]. The predicted σ^70^-dependent -10 and -35 sequences are underlined. The predicted translation start codon of *gadC* is in bold italics. (**C**) **Quantitative real-time RT-PCR.** Quantification of *FTN_0570* expression in *F. novicida* strain U112 (WT) or *ΔgadC* mutant were performed in TSB at 37°C. qRT-PCRs were performed twice using independent samples (in triplicate).
(TIFF)

Figure S2 **Growth kinetics in broth.** Stationary-phase bacterial cultures of wild-type *F. novicida* and Δ*gadC* mutant strains were diluted to a final OD_600_ of 0.1, in 20 mL broth. Every hour, the OD_600_ of the culture was measured, during a 9 h-period. (**A**) CDM, chemically defined medium; (**B**) TSB, tryptic soy broth.
(TIFF)

Figure S3 **Cell death.** The cell death kinetics of infected BMM (from BALB/c mice) was followed by monitoring propidium iodide (PI) incorporation in real time. PI fluorescence was measured every 15 min on a microplate fluorimeter (Tecan Infinite 1000). BMM were infected with wild-type *F. novicida*, (WT), the Δ*gadC* mutant (Δ*gadC*), or the Δ*FPI* mutant (Δ*FPI*).
(TIFF)

Figure S4 **The decarboxylase mutant Δ** ***gadB*** **.** (**A**) Intracellular replication of wild-type *F. novicida* carrying the empty plasmid pKK214 (WT/pKK(−)), of the mutant Δ*gadB* and complemented strain (Δ*gadB*/pKK-*gadB*), and of the Δ*FPI* mutant (Δ*FPI*), was monitored in J774.1 macrophage-like cells over a 24 h-period. Results are shown as the average of log_10_ (cfu mL^−1^) ± standard deviation. (**B**) Competition assays were performed by infecting a group of five female BALB/c mice by the i.p. route with a 1∶1 mixture of wild-type bacteria and Δ*gadB* mutant strain (100 cfu of each). The data represent the competitive index (CI) value for cfu of mutant/wild-type in the liver (L: black diamonds, left column) and spleen (S: black circles, right column) of each mouse, 48 h after infection. Bars represent the geometric mean CI value.
(TIFF)

Figure S5 **Oxidative stress response in the presence of the TCA cycle intermediates.** Exponential phase bacteria, diluted in chemically defined medium supplemented with 1.5 mM glutamate were subjected to oxidative stress (500 µM H_2_0_2_). *Upper panel*: in the presence or absence of fumarate (1.5 mM); *middle panel*: in the presence or absence of succinate (1.5 mM); *lower panel*: in the presence or absence of oxoglutarate (1.5 mM). The bacteria were plated on chocolate agar plates at different times and viable bacteria were monitored 2 days after. Data are the average cfu mL^−1^ for three points. Experiments were realized twice. **, *p*<0.01 (as determined by the Student's *t*-test).
(TIFF)

Figure S6 **Fate of GadC-dependent glutamate entry into** ***Francisella*** **.** External glutamate (Glu_E_) is taken up by the GadC permease. Internal Glutamate (Glu_I_) can be converted either to: i) glutamine by the glutamine synthase GlnA; ii) GABA by the glutamate decarboxylase GadD; ii) oxoglutarate (OG) by the glutamate dehydrogenase GdhA; or iii) glutathione (GSH) by the glutamate-cysteine ligase GshA and the glutathione synthetase GshB. Internal GABA may be either: i) translocated out of the cytoplasm, through GadC; or ii) converted to succinate (S), via the GABA shunt. The dotted green arrow indicates the alternative pathway leading (from Glu_I_) to glutathione production (GSH, reduced form, GSSG, oxidized form). The dotted red arrows indicate the two possible pathways leading to the tricarboxylic acid (TCA) cycle: i) from glutamate to oxoglutarate, or ii) from GABA to succinate. OA, oxaloacetate; C, citrate; A, cis-aconitate; IC, isocitrate; OS, oxalosuccinate; S-CoA, succinyl-CoA; F, fumarate; M, malate. The two anti-oxidant pathways (GshA/GshB in green; GdhA in pink) lead to the production of glutathione (GSH) and oxoglutarate (OG) + NADPH, respectively. They allow the oxidoreduction reactions: 2 GSH + ROOH ↔ GSSG + ROH + H_2_O; and NADPH + 2O_2_ ↔ NADP^+^ + 2O_2_^−^ + H^+^), respectively.
(TIFF)

Text S1 **Supporting text.** This file includes one Table listing the strains, plasmids and primers used in this study (Table S1), Supplemental Experimental Procedures, and Supplemental References.
(DOCX)
