# Supplementary figures and images for: Glutamate Utilization Couples Oxidative Stress Defense and the Tricarboxylic Acid Cycle in Francisella Phagosomal Escape
Source: PLoS Pathog. 2014 Jan 16;10(1):e1003893. doi: 10.1371/journal.ppat.1003893 (PMC3894225; doi:10.1371/journal.ppat.1003893)

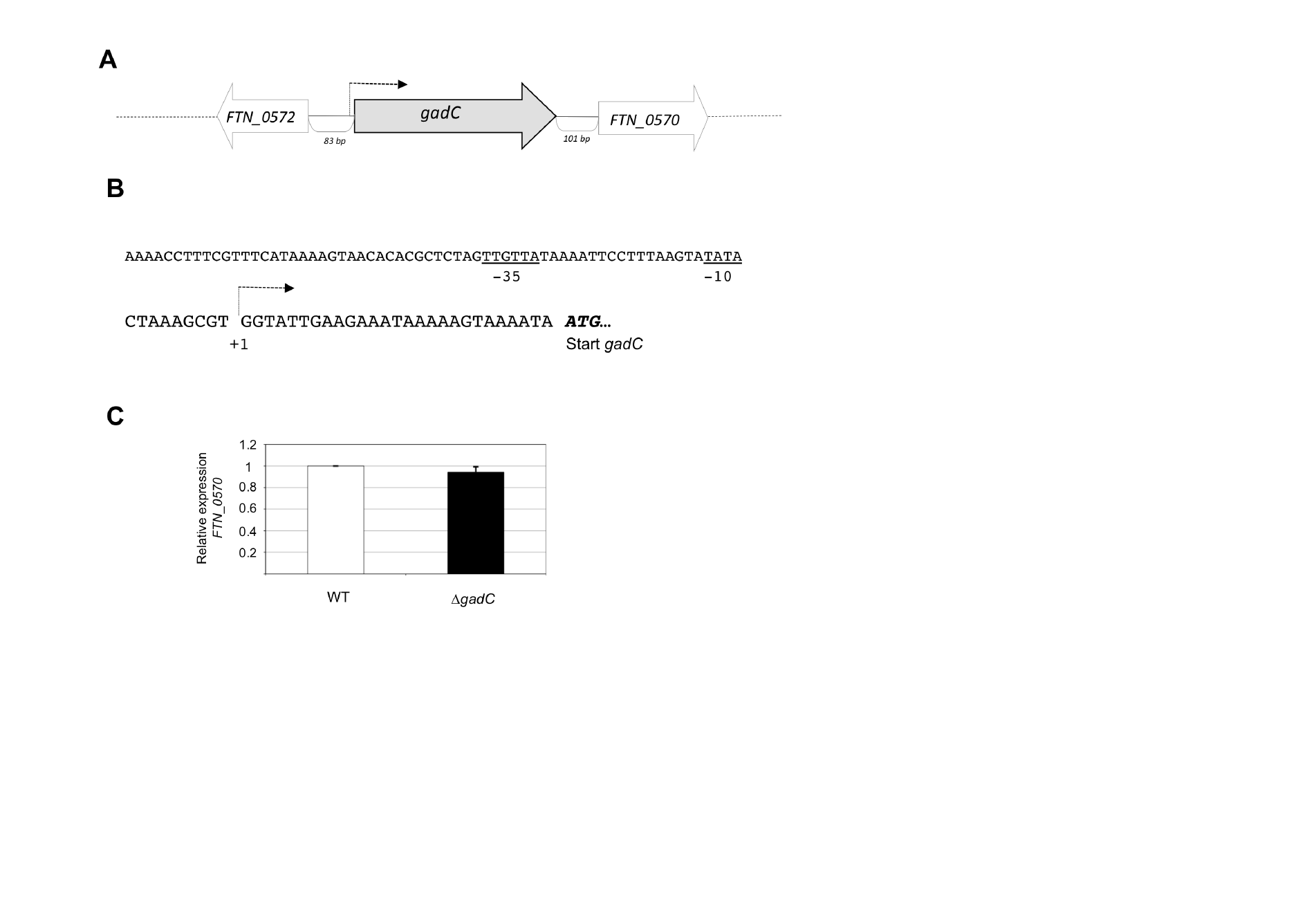

Supplement: Figure S1 — The gadC region. (A) Schematic organization. The gene gadC (FTN_0571, grey arrow) is flanked, upstream (83 bp) by gene FTN_0572 (transcribed on the opposite strand); and downstream, by gene FTN_0570 (white arrows), separated by a 101 bp intergenic region. (B) Transcriptional analysis. We performed rapid amplification of cDNA ends (5′-RACE) to determine the 5′ end of the gadC mRNA. A broken arrow shows the transcription start of gadC (+1). Inspection of the sequence immediately upstream of the transcriptional start identified putative -10 and -35 promoter elements that share homology to the consensus site recognized by the major sigma factor σ70 [52]. The predicted σ70-dependent -10 and -35 sequences are underlined. The predicted translation start codon of gadC is in bold italics. (C) Quantitative real-time RT-PCR. Quantification of FTN_0570 expression in F. novicida strain U112 (WT) or ΔgadC mutant were performed in TSB at 37°C. qRT-PCRs were performed twice using independent samples (in triplicate). (TIFF) [file ppat.1003893.s001.tif]

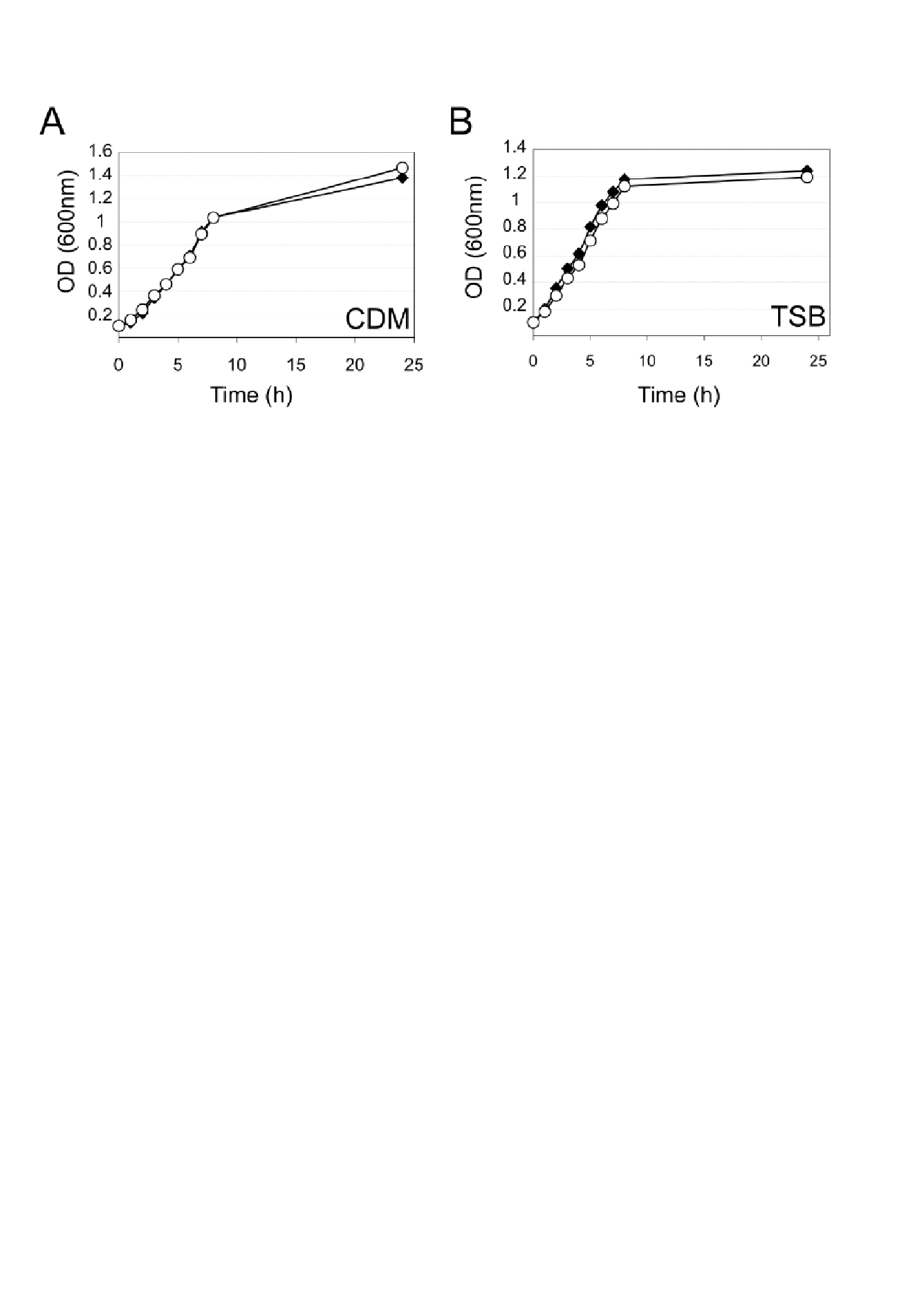

Supplement: Figure S2 — Growth kinetics in broth. Stationary-phase bacterial cultures of wild-type F. novicida and ΔgadC mutant strains were diluted to a final OD600 of 0.1, in 20 mL broth. Every hour, the OD600 of the culture was measured, during a 9 h-period. (A) CDM, chemically defined medium; (B) TSB, tryptic soy broth. (TIFF) [file ppat.1003893.s002.tif]

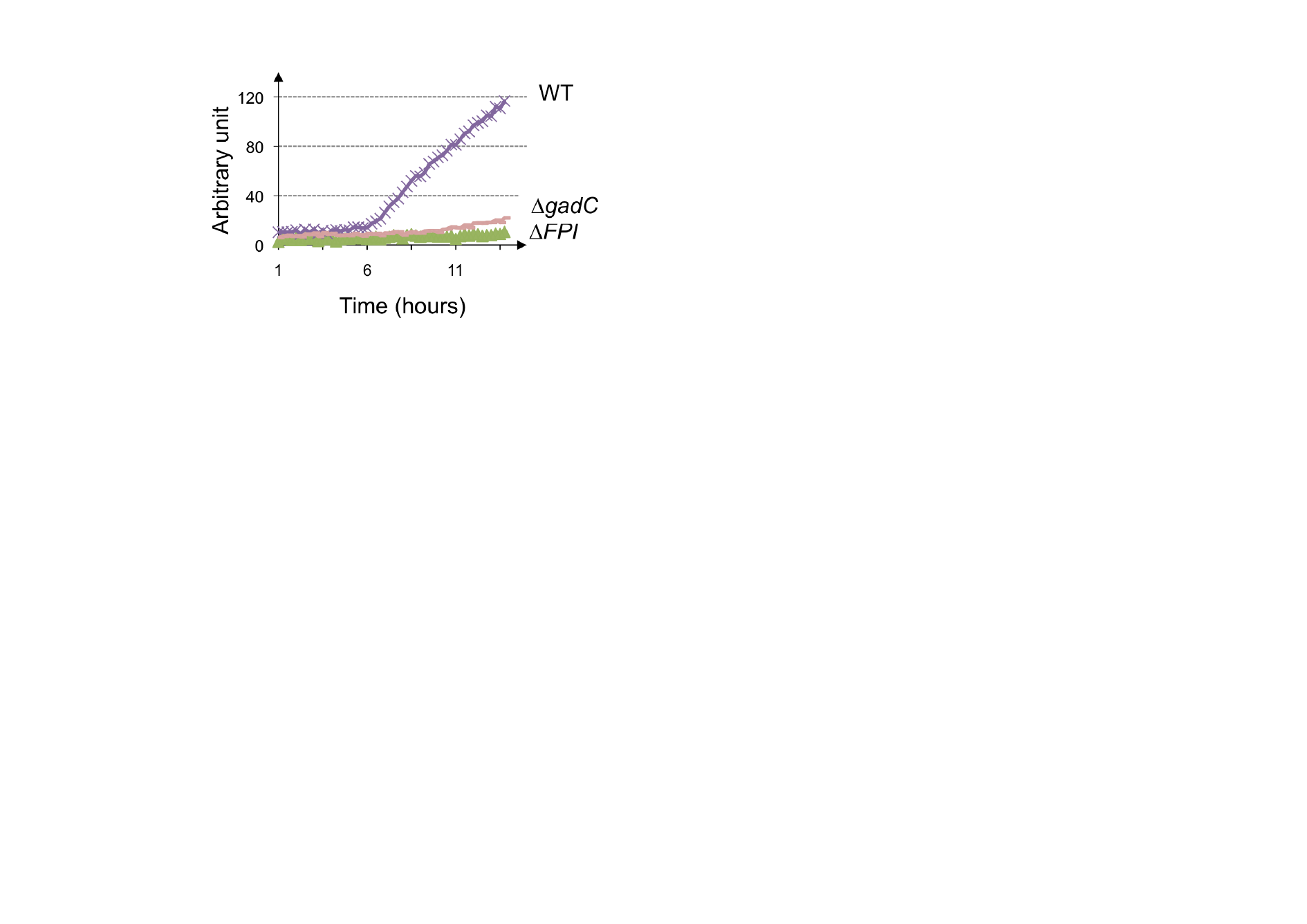

Supplement: Figure S3 — Cell death. The cell death kinetics of infected BMM (from BALB/c mice) was followed by monitoring propidium iodide (PI) incorporation in real time. PI fluorescence was measured every 15 min on a microplate fluorimeter (Tecan Infinite 1000). BMM were infected with wild-type F. novicida, (WT), the ΔgadC mutant (ΔgadC), or the ΔFPI mutant (ΔFPI). (TIFF) [file ppat.1003893.s003.tif]

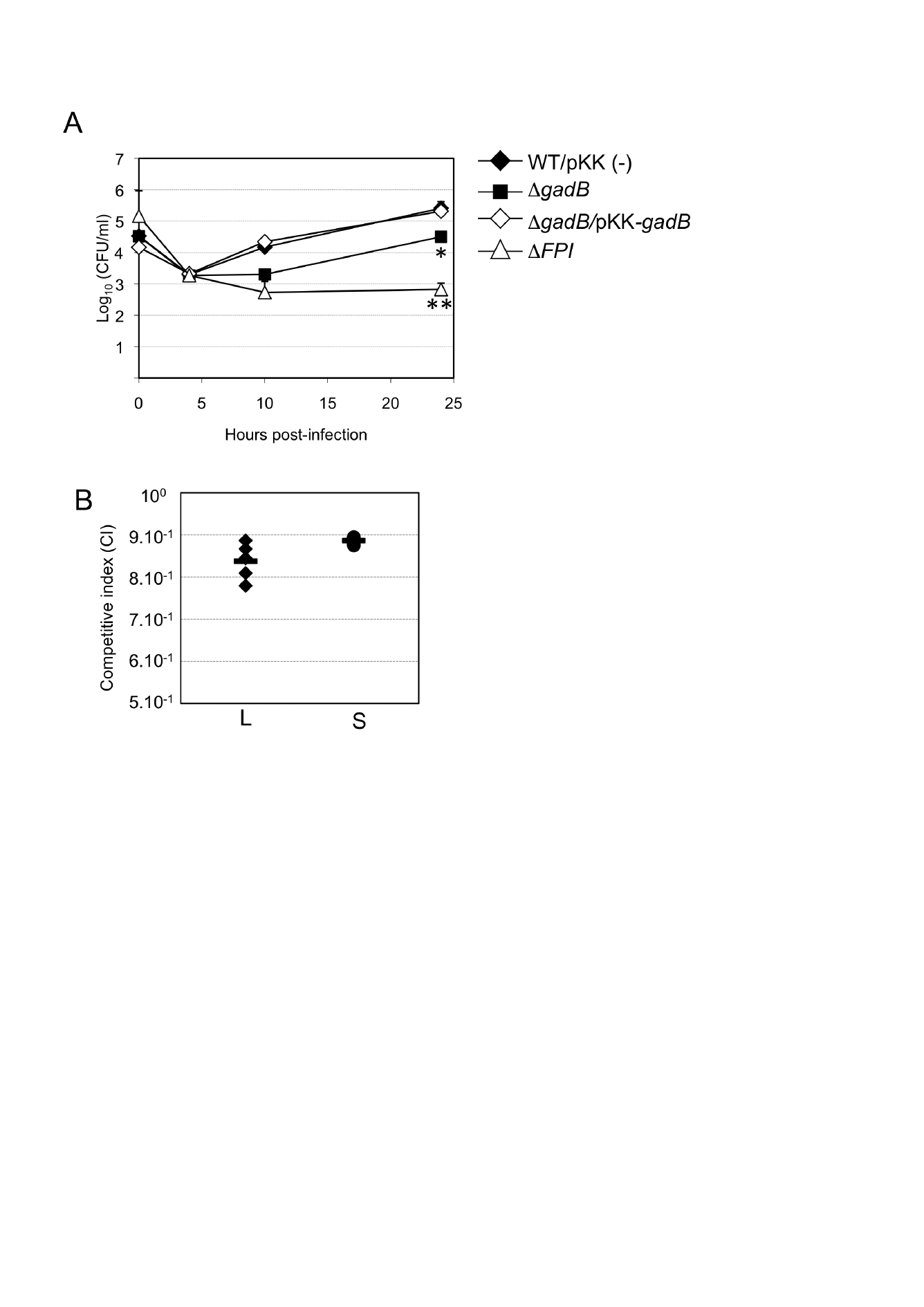

Supplement: Figure S4 — The decarboxylase mutant Δ gadB . (A) Intracellular replication of wild-type F. novicida carrying the empty plasmid pKK214 (WT/pKK(−)), of the mutant ΔgadB and complemented strain (ΔgadB/pKK-gadB), and of the ΔFPI mutant (ΔFPI), was monitored in J774.1 macrophage-like cells over a 24 h-period. Results are shown as the average of log10 (cfu mL−1) ± standard deviation. (B) Competition assays were performed by infecting a group of five female BALB/c mice by the i.p. route with a 1∶1 mixture of wild-type bacteria and ΔgadB mutant strain (100 cfu of each). The data represent the competitive index (CI) value for cfu of mutant/wild-type in the liver (L: black diamonds, left column) and spleen (S: black circles, right column) of each mouse, 48 h after infection. Bars represent the geometric mean CI value. (TIFF) [file ppat.1003893.s004.tif]

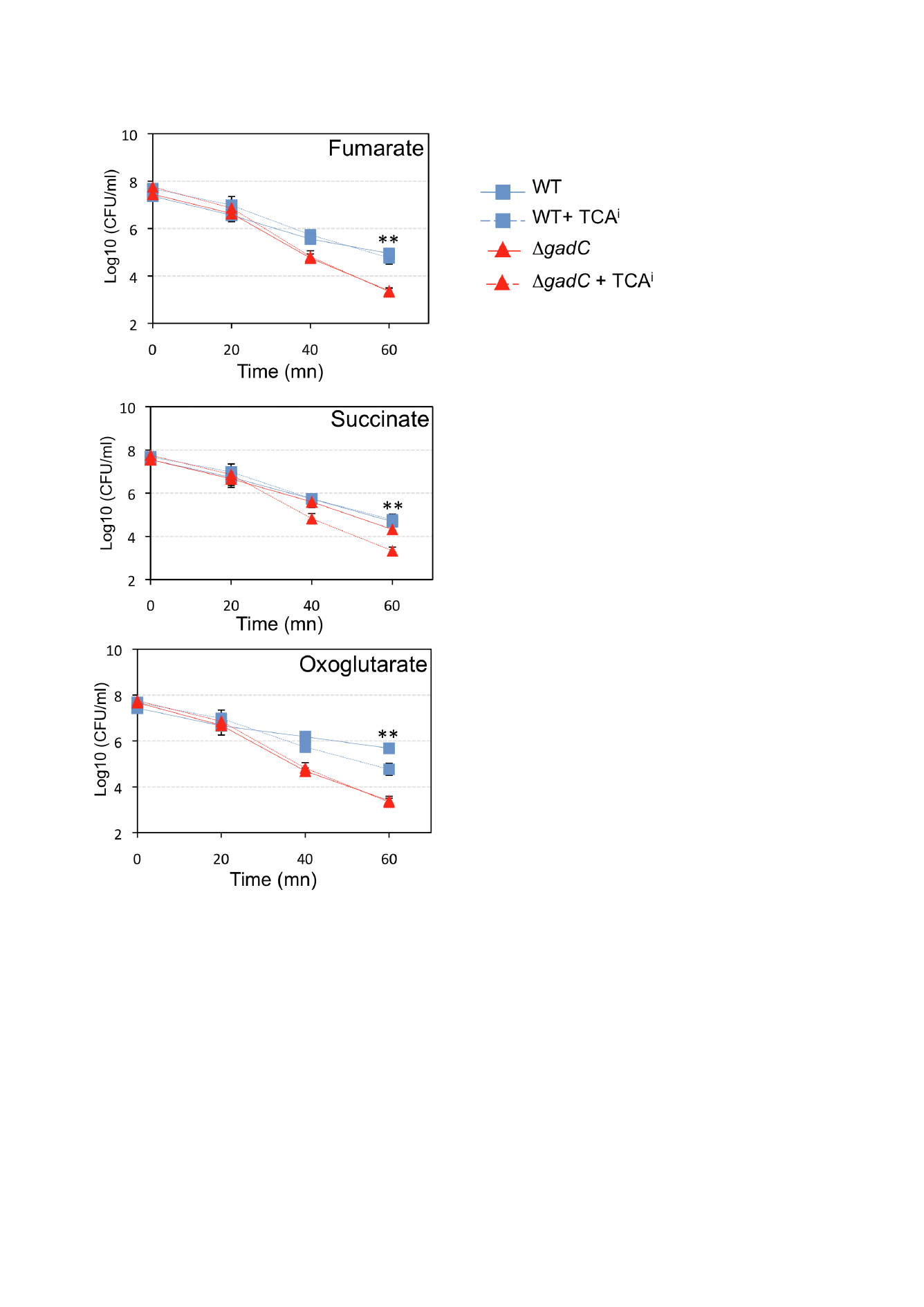

Supplement: Figure S5 — Oxidative stress response in the presence of the TCA cycle intermediates. Exponential phase bacteria, diluted in chemically defined medium supplemented with 1.5 mM glutamate were subjected to oxidative stress (500 µM H202). Upper panel: in the presence or absence of fumarate (1.5 mM); middle panel: in the presence or absence of succinate (1.5 mM); lower panel: in the presence or absence of oxoglutarate (1.5 mM). The bacteria were plated on chocolate agar plates at different times and viable bacteria were monitored 2 days after. Data are the average cfu mL−1 for three points. Experiments were realized twice. **, p<0.01 (as determined by the Student's t-test). (TIFF) [file ppat.1003893.s005.tif]

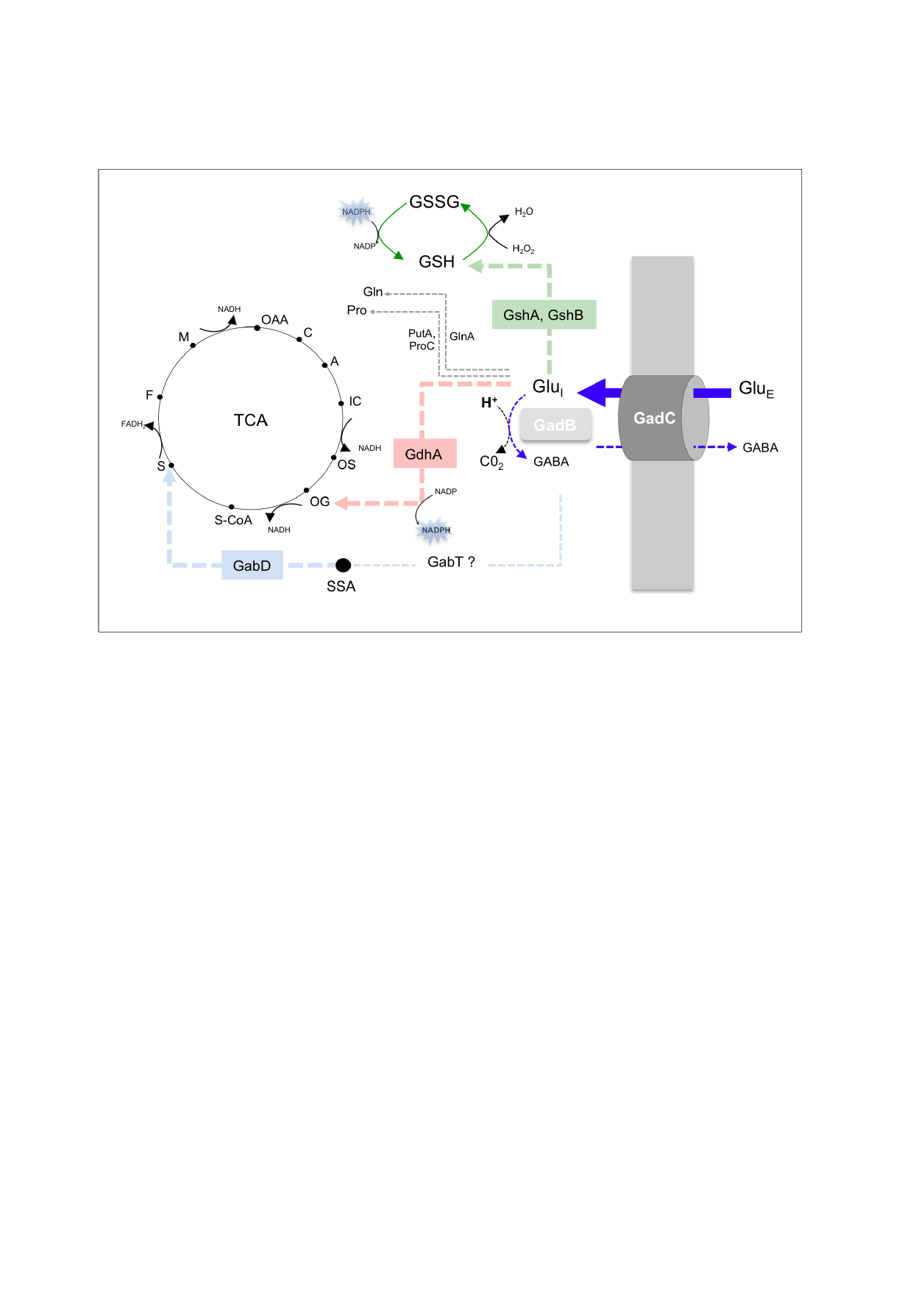

Supplement: Figure S6 — Fate of GadC-dependent glutamate entry into Francisella . External glutamate (GluE) is taken up by the GadC permease. Internal Glutamate (GluI) can be converted either to: i) glutamine by the glutamine synthase GlnA; ii) GABA by the glutamate decarboxylase GadD; ii) oxoglutarate (OG) by the glutamate dehydrogenase GdhA; or iii) glutathione (GSH) by the glutamate-cysteine ligase GshA and the glutathione synthetase GshB. Internal GABA may be either: i) translocated out of the cytoplasm, through GadC; or ii) converted to succinate (S), via the GABA shunt. The dotted green arrow indicates the alternative pathway leading (from GluI) to glutathione production (GSH, reduced form, GSSG, oxidized form). The dotted red arrows indicate the two possible pathways leading to the tricarboxylic acid (TCA) cycle: i) from glutamate to oxoglutarate, or ii) from GABA to succinate. OA, oxaloacetate; C, citrate; A, cis-aconitate; IC, isocitrate; OS, oxalosuccinate; S-CoA, succinyl-CoA; F, fumarate; M, malate. The two anti-oxidant pathways (GshA/GshB in green; GdhA in pink) lead to the production of glutathione (GSH) and oxoglutarate (OG) + NADPH, respectively. They allow the oxidoreduction reactions: 2 GSH + ROOH ↔ GSSG + ROH + H2O; and NADPH + 2O2 ↔ NADP+ + 2O2 − + H+), respectively. (TIFF) [file ppat.1003893.s006.tif]
