# Supplementary material for: Glutamate Utilization Couples Oxidative Stress Defense and the Tricarboxylic Acid Cycle in Francisella Phagosomal Escape
Source: PLoS Pathog. 2014 Jan 16;10(1):e1003893. doi: 10.1371/journal.ppat.1003893 (PMC3894225; doi:10.1371/journal.ppat.1003893)
Supplement: Text S1 — Supporting text. This file includes one Table listing the strains, plasmids and primers used in this study (Table S1), Supplemental Experimental Procedures, and Supplemental References. (DOCX) [file ppat.1003893.s007.docx]

**Table S1. Strains, plasmids and primers**

| Strain, plasmid, or primer | Primer code | Description or sequence (5’ → 3’) | Reference or source |
| --- | --- | --- | --- |
| *E. coli* strains |  |  |  |
| *E. coli* DH5α |  | F– Φ80*lac*ZΔM15 Δ(*lac*ZYA-*arg*F) U169 *rec*A1 *end*A1 *hsd*R17 (rK–, mK+) *pho*A *sup*E44 λ– *thi*-1 *gyr*A96 *rel*A1 | Laboratory strain collection |
| *E. coli* Top10 |  | F– *mcr*A Δ(*mrr*-*hsd*RMS-*mcr*BC) Φ80*lac*ZΔM15 Δ*lac*X74 *rec*A1*ara*D139 Δ(*ara leu*) 7697 *gal*U *gal*K *rps*L (StrR) *end*A1 *nup*G | Laboratory strain collection |
| EF491 |  | *E. coli* K12 *gadC*::pRR10 (Ap) | J.W. Foster (Castanie-Cornet et al., 1999) |
| EF547 |  | *E. coli* K12 *gadC*::pRR10 (Ap)/pCF348 (*gadC*+ Tc^R^) | J.W. Foster (Castanie-Cornet et al., 1999) |
| EF491 pCR.1TOPO*-FTN_0571* |  | *E. coli* K12 *gadC*::pRR10 (Ap) containing plasmid pCR.1TOPO-*FTN_0571* | This study |
|  |  |  |  |
| *F. tularensis* strains |  |  |  |
| U112 |  | *F. tularensis* subsp. *novicida* U112 | Laboratory strain collection |
| FTN (PKK214) |  | FTN containing empty plasmid PKK214 | This study |
| FTNΔ*FTN_0571* |  | FTN with gene *FTN_0571* deleted | This study |
| FTNΔ*FTN_0571* (PKK214-*FTN_0571)* |  | FTN*ΔFTN_0571* containing PKK214-*FTN_0571* | This study |
| FTNΔ*FTN_1701* |  | FTN with gene *FTN_1701* deleted | This study |
| FTNΔ*FTN_1701* (PKK214-*FTN_1701)* |  | FTNΔ*FTN_1701* containing PKK214-*FTN_1701* | This study |
|  |  |  |  |
| Plasmids |  |  |  |
| pKK214 |  | Derived from pKK202, promoter trap vector drives Cm^R^ Tet^R^ | Laboratory plasmid collection |
| pCR2.1TOPO |  | PCR cloning vector, Amp^R^ Km^R^ | Invitrogen |
|  |  |  |  |
| Primers |  |  |  |
| Pgro_F | 1 | TTG TAT GGA TTA GTC GAG CTA AA |  |
| npt_R | 2 | TCA GAA GAA CTC GTC AAG AAG G |  |
| FTN_0571upF | 3 | CAC ACC TTT ACC TAG TCT TTG C |  |
| FTN_0571upR | 4 | GAG CTT TTT AGC TCG ACT AAT CCA TAC AAC TTA AGC TAT CGA CAG CGA T |  |
| FTN_0571downF | 5 | CTA TCG CCT TCT TGA CGA GTT CTT CTG ACG ATA TGAA TGC TTA TTG TAG G |  |
| FTN_0571downR | 6 | GTC GAT ACT ATC AAA CCA GC |  |
| FTN_0571upF2 control | 7 | ACA TGG AGC TAT TTG TTT GA |  |
| FTN_0571downR2 control | 8 | TTA GTA CAG AAA ATA AAA CTG G |  |
| FTN_0571 5'race GSP1 | 9 | CGA AGA ATC CAC AAA TAG ATG G |  |
| FTN_0571 5'race GSP2 | 10 | ACT TTG TAA ACC CAC TGT AAC C |  |
| FTN_0571 compl forw | 11 | GAA TCC TTT AGA GTG TCA AAG CTT TT |  |
| FTN_0571 compl rev | 12 | CAT ACT ACT GAA ATT GTT GCG CC |  |
| FTN_0571 qRT forw | 13 | CAT ACT ACT GAA ATT GTT GCG CC |  |
| FTN_0571 qRT rev | 14 | CCT GAA ATT AGG AGG GCA CGA |  |
| FTN_0570 qRT forw | 15 | GGT GAT TAT ATA GCT CCA ATA TTA GCT GCA |  |
| FTN_0570 qRT rev | 16 | AAT ATA TAC TAA AAC TAT ACG CTT AAG CTT GGT G |  |
| FTN_0571up PCR TOPO | 17 | CGTTTCATAAAAGTAACACACGCTCTAG |  |
| FTN_0571down PCR TOPO | 18 | GAA TTC AAA GTG TTG ATA TTG TAA CAG TTT TAC |  |
| FTN_1701upF | 19 | TCA AGA GAT TGG TGC GGA T |  |
| FTN_1701upR | 20 | GAG CTT TTT AGC TCG ACT AAT CCA TAC AAT CAT CAA CTT CCG TTT GGC |  |
| FTN_1701downF | 21 | CTA TCG CCT TCT TGA CGA GTT CTT CTG ATG ATG ATT AGA GAT TTA GTT GCG G |  |
| FTN_1701downR | 22 | TGG CAC ATT AGT GAG ATT GCC |  |
| FTN_1701upF2 control | 23 | AGA GGA TAT CTT TGT TGC AGA AA |  |
| FTN_1701downR2 control | 24 | CAT AGC CCC CTT TGA CAT G |  |
| FTN_1701 compl forw | 25 | CCC GGG CTC ATG GCT TAG TAA TAG TTA TCC |  |
| FTN_1701 compl rev | 26 | GAA TTC AAC CTG ATC TGG TCC AGC |  |
| FTN_1700 qRT forw | 27 | GCA GGG ATT GCA ACT ATG GAT C |  |
| FTN_1700 qRT rev | 28 | ATA TCT AAC ATG ACC GAT ACC CAT ATT ACC |  |
| FTN_1701 qRT forw | 29 | TCG AAG CAA AAT TTA GCG ACG |  |
| FTN_1701 qRT rev | 30 | GTT TGC CAG GAT ATT T |  |
| FTN_1702 qRT forw | 31 | GCT GAT GAA GTC GCG ATG GTT GGC G |  |
| FTN_1702 qRT rev | 32 | CGG CAC CGT TTC AAC ATT TGC CGC |  |
| FTN_1532 qRT forw | 33 | AGG CTT CGA GCA AGT TTT CA |  |
| FTN_1532 qRT rev | 34 | ACC GCA CCA TAA CCT GTA GC |  |
| FTN_0127 qRT forw | 35 | AGC CAT TAG CTG AGG CAA AA |  |
| FTN_0127 qRT rev | 36 | TGC AAG TGC TGT GAG AGG AG |  |
| FTN_0277 qRT forw | 37 | CAA GTC CCG CAC AAA AAG AT |  |
| FTN_0277 qRT rev | 38 | CTT ATA CCC ACC GGC TCA AA |  |
| FTN_0804 qRT forw | 39 | CGC ATA CCT TGA TCA CCA GA |  |
| FTN_0804 qRT rev | 40 | CTG CAA GAT TGC CAC GAT TA |  |
| FTN_0593 qRT forw | 41 | CGT GGC GTA ACT CCT GGT AA |  |
| FTN_0593 qRT rev | 42 | TGA CCC GGC ATA ATA CCA AT |  |
| FTN_1434 qRT forw | 43 | TTG TTT GGT CAT GCG GTA GA |  |
| FTN_1434 qRT rev | 44 | TTT GCC ATC TTT GTT CCA CA |  |
| FTN_1635 qRT forw | 45 | GCT CAA TTT GGC GAT TTT GT |  |
| FTN_1635 qRT rev | 46 | TCG GCG TCA TTA CAA TCA AA |  |

**Supplemental Experimental procedures**

**Bacterial growth**

*F. novicida* and its mutant derivatives were grown: i) in liquid, on Tryptic Soya broth (TSB, Becton, Dickinson and company) or Chamberlain chemically defined medium (CDM) and ii) in solid, on pre-made chocolate agar PolyViteX (BioMerieux SA Marcy l’Etoile, France) or chocolate plates prepared from GC medium base, IsoVitalex vitamins and haemoglobin (BD Biosciences, San Jose, CA, USA), at 37°C. *E. coli* was grown in LB (Luria-Bertani, Difco) at 37°C. Ampicillin was used at a final concentration of 100 µg mL^-1^ to select recombinant *E. coli* carrying pGEM and its derivatives. Kanamycin was used at a final concentration of 50 µg mL^-1^ and 20 µg mL^-1^ to select respectively recombinant *E. coli* and *Francisella* carrying pKK and its derivates. All bacterial strains, plasmids, and primers used in this study are listed in **Supplemental Table 1**.

**Construction of a chromosomal ∆*gadC* deletion mutant**

We have generated a chromosomal deletion of gene *FTN_0571* (*gadC*) in *F. novicida* strain U112 by allelic replacement of the wild-type region with a mutated region deleted of the entire *gadC* gene (from the ATG start codon till the TAA stop codon), substituted by the kanamycine resistance gene *npt* placed under the control of the P*gro* promoter. First, the two regions (app. 600 bp each) flanking gene *gadC* (designated *FTN_0571*up and *FTN_0571*down, respectively), and the *npt* gene (1,161 bp, amplified from plasmid pFNLTP16H3, (Maier et al., 2006), were amplified by PCR using the following pairs of primers: i) *FTN_0571*up, p3, p4; ii) *FTN_0571*down, p5, p6; iii) *npt*, p1, p2. The region *FTN_0571*up*-npt-FTN_0571*down (*ca.* 2300 bp) was then amplified by triple overlap PCR, using the *FTN_0571*up, *FTN_0571*down and *npt* products. The resulting PCR product was gel purifed (using the QIAquick Gel extraction kit, QIAgen) and directly used to transform wild-type *F. novicida*. Chemical transformation was performed as described in (Ludu et al., 2008). Recombinant bacteria, resulting from allelic replacement of the wild-type region with the mutated *FTN_0571up/npt/ FTN_0571down* region, were selected on kanamycine-containing plates (20 µg mL^-1^ ). The mutant strain, designated ∆*gadC,* was checked for loss of the wild-type *gadC* gene, using specific primers in PCR and qRT_PCR, by PCR sequencing (GATC Biotech) and Southern blot.

**Functional complementation**

The plasmid used for complementation of the ∆*gadC* mutant, pKK-*gadC*, was constructed by amplifying a 1,588 bp fragment (corresponding to the sequence 148 bp upstream of the *gadC* start codon and to 25 bp downstream of the stop codon) using primers *FTN_0571* compl forw and *FTN_0571* compl rev (**Supplemental Table 1**), followed by digestion with *Eco*RI and *Sma*I, and cloning into plasmid pKK214 (Kuoppa et al., 2001).

The plasmid used for complementation of the *∆gadB* mutant, pKK-*gadB*, was constructed by amplifying a 1,638 bp fragment (corresponding to the sequence 159 bp upstream of the *gadC* start codon and to 133 bp downstream of the stop codon) using primers FTN_1701 compl forw and FTN_1701 compl rev (**Supplemental Table 1**), followed by digestion with *Eco*RI and *Sma*I, and cloning into plasmid pKK214 (Kuoppa et al., 2001). The plasmids pKK214, pKK214-*gadB* and pKK214-*gadC,* were introduced into wild-type *F. novicida* or into the ∆*gadB* and ∆*gadC* mutants by electroporation, as described previously (Dieppedale et al., 2011).

**Multiplication in macrophages**

J774.1 macrophage-like cells (ATCC Number: TIB67) were propagated in Dulbecco´s Modified Eagle's Medium (DMEM) containing 10% fetal calf serum, whereas human monocyte-like cell line THP-1 (ATCC Number: TIB202) and bone marrow-derived macrophages (BMM) from BALB/c were propagated in RPMI Medium 1640 containing 10% fetal calf serum, respectively. J774.1 and BMM were seeded at a concentration of ~2 x 10^5^ cells per well in 12-well cell tissue plates and monolayers were used 24 h after seeding. THP-1 were seeded at a concentration of ~2 x 10^5^ cells per well in 12-well cell tissue plates 48h before infection, and supplemented with phorbol myristate acetate (PMA) to induce cell differentiation (200 ng mL^-1^). J774.1, BMM and THP-1 were incubated for 60 min at 37°C with the bacterial suspensions (approximate multiplicities of infection 100) to allow the bacteria to enter. After washing (time zero of the kinetic analysis), the cells were incubated in fresh culture medium containing gentamicin (10 μg mL^-1^) to kill extracellular bacteria. At several time-points, cells were washed three times in DMEM or RPMI, macrophages were lysed by addition of water and the titer of viable bacteria released from the cells was determined by spreading preparations on Chocolate agar plates. For each strain and time in an experiment, the assay was performed in triplicate. Each experiment was independently repeated at least three times and the data presented originate from one typical experiment.

**Real time cell death assay**

The cell death kinetics was followed by monitoring propidium iodide incorporation in real time. Briefly, 5x10^4^ BMM were seeded in 0.3 cm^2^ wells of black 96 flat-bottom-well plate 24 hours before infection. Infection was performed as described above. One hour post-infection, cells were washed three times and placed in CO2-independent medium (Gibco) supplemented with propidium iodide (5 µg mL^-1^), 10% FCS, 10% M-CSF containing supernatant. Propidium iodide fluorescence was measured every 15 minutes on a micro plate fluorimeter (Tecan Infinite 1000).

**References**

Alkhuder, K., Meibom, K.L., Dubail, I., Dupuis, M., and Charbit, A. (2009). Glutathione provides a source of cysteine essential for intracellular multiplication of *Francisella tularensis*. PLoS Pathog *5*, e1000284.

Castanie-Cornet, M.P., Penfound, T.A., Smith, D., Elliott, J.F., and Foster, J.W. (1999). Control of acid resistance in *Escherichia coli*. J Bacteriol *181*, 3525-3535.

Dieppedale, J., Sobral, D., Dupuis, M., Dubail, I., Klimentova, J., Stulik, J., Postic, G., Frapy, E., Meibom, K.L., Barel, M.*, et al.* (2011). Identification of a putative chaperone involved in stress resistance and virulence in *Francisella tularensis*. Infect Immun *79*, 1428-1439.

Kuoppa, K., Forsberg, A., and Norqvist, A. (2001). Construction of a reporter plasmid for screening in vivo promoter activity in *Francisella tularensis*. FEMS Microbiol Lett *205*, 77-81.

Ludu, J.S., de Bruin, O.M., Duplantis, B.N., Schmerk, C.L., Chou, A.Y., Elkins, K.L., and Nano, F.E. (2008). The Francisella pathogenicity island protein PdpD is required for full virulence and associates with homologues of the type VI secretion system. J Bacteriol *190*, 4584-4595.

Maier, T.M., Pechous, R., Casey, M., Zahrt, T.C., and Frank, D.W. (2006). In vivo Himar1-based transposon mutagenesis of *Francisella tularensis*. Appl Environ Microbiol *72*, 1878-1885.
